# Trauma in Neonatal Acute Brain Slices Alters Calcium and Network Dynamics and Causes Calpain-Mediated Cell Death

**DOI:** 10.1523/ENEURO.0007-24.2024

**Published:** 2024-07-04

**Authors:** Pratyush Suryavanshi, Samuel Baule, Joseph Glykys

**Affiliations:** ^1^Department of Pediatrics, The University of Iowa, Iowa City, Iowa 52241; ^2^Iowa Neuroscience Institute, The University of Iowa, Iowa City, Iowa 52241; ^3^Departments of Biomedical Engineering, The University of Iowa, Iowa City, Iowa 52241; ^4^Neurology, The University of Iowa, Iowa City, Iowa 52241

**Keywords:** calcium overload, GCaMP, neuronal death, somatosensory, trauma

## Abstract

Preparing acute brain slices produces trauma that mimics severe penetrating brain injury. In neonatal acute brain slices, the spatiotemporal characteristics of trauma-induced calcium dynamics in neurons and its effect on network activity are relatively unknown. Using multiphoton laser scanning microscopy of the somatosensory neocortex in acute neonatal mouse brain slices (P8–12), we simultaneously imaged neuronal Ca^2+^ dynamics (GCaMP6s) and cytotoxicity (propidium iodide or PI) to determine the relationship between cytotoxic Ca^2+^ loaded neurons (GCaMP-filled) and cell viability at different depths and incubation times. PI^+^ cells and GCaMP-filled neurons were abundant at the surface of the slices, with an exponential decrease with depth. Regions with high PI^+^ cells correlated with elevated neuronal and neuropil Ca^2+^. The number of PI^+^ cells and GCaMP-filled neurons increased with prolonged incubation. GCaMP-filled neurons did not participate in stimulus-evoked or seizure-evoked network activity. Significantly, the superficial tissue, with a higher degree of trauma-induced injury, showed attenuated seizure-related neuronal Ca^2+^ responses. Calpain inhibition prevented the increase in PI^+^ cells and GCaMP-filled neurons in the deep tissue and during prolonged incubation times. Isoform-specific pharmacological inhibition implicated calpain-2 as a significant contributor to trauma-induced injury in acute slices. Our results show a calpain-mediated spatiotemporal relationship between cell death and aberrant neuronal Ca^2+^ load in acute neonatal brain slices. Also, we demonstrate that neurons in acute brain slices exhibit altered physiology depending on the degree of trauma-induced injury. Blocking calpains may be a therapeutic option to prevent acute neuronal death during traumatic brain injury in the young brain.

## Significance Statement

This is the first study to characterize the spatiotemporal dynamics of neocortical injury in acute neonatal slices mimicking severe penetrating traumatic brain injury, using PI labeling and elevated neuronal Ca^2+^ load as markers for cytotoxicity. We found depth- and time-dependent neuronal damage, leading to altered neuronal responses. Elevate neuronal Ca^2+^ and cytotoxicity were mitigated by pharmacologically inhibiting calpains, a family of Ca^2+^-dependent proteases involved in multiple cell death mechanisms. Our study provides evidence for injury-dependent neuronal and circuit function alterations in neonatal acute brain slices. Calpain inhibition decreased trauma-induced cell death in the neonatal brain, identifying them as potential therapeutic targets at this age.

## Introduction

Acute brain slices are widely used in neuroscience and have been instrumental in gaining insights into neuroglial function at molecular, synaptic, and cellular levels. Brain slices are well suited for various experimental approaches like electrophysiology, fluorescent imaging, and biochemical and histological techniques (Yamamoto and McIlwain, 1966; Collingridge, 1995; Colbert, 2006). However, the preparation and incubation of the brain slices result in trauma and incubation-dependent cellular injury, mimicking penetrating brain injury. Here, we evaluated the spatiotemporal dynamics of cell death, its correlation with neuronal Ca^2+^ overload, and the role of calpains in neonatal neocortical brain slices.

Slice preparation, which can be thought of as a model of severe brain injury, induces long-term morphological changes, including neuronal swelling, dendritic beading (Kirov et al., 2004; Greenwood et al., 2007; Liang et al., 2007), and alterations in synapse density, signaling, and chloride homeostasis, especially in the superficial tissue (Kirov et al., 1999; Davies et al., 2007; Dzhala et al., 2012; Glykys and Staley, 2016). Prolonged incubation of brain slices can also alter pH, temperature, oxygenation, and glucose levels with time (Schurr et al., 1985; Reid et al., 1988). Importantly, trauma during brain slice preparation can also cause a long-lasting elevation in neuronal [Ca^2+^], deleterious to neuronal health (Berridge et al., 2003; Szydlowska and Tymianski, 2010).

Multiple studies on cultured cells report that injured neurons with high Ca^2+^ load often exhibit abnormal nuclear accumulation of otherwise cytosolic GCaMP (Bano et al., 2010; Strasser et al., 2012). Also, Ca^2+^-activated and calpain-mediated processes increase neuronal nuclear membrane permeability, facilitating nuclear translocation of cytosolic GCaMP6s (Bano et al., 2010; Suryavanshi et al., 2024). This nuclear accumulation and Ca^2+^ overload impart a “filled” appearance to the injured neuron (Yang et al., 2018). However, contributions of [Ca^2+^]/calpain-mediated processes in trauma-related injury of acute brain slices remain unexplored, especially in the neonatal brain.

In this manuscript, we evaluated the relationship between trauma-induced Ca^2+^ load in neurons and the neuropil with cell death across different tissue depths in acute neonatal brain slices and during several incubation time points. Additionally, we investigated the role of calpains, a family of Ca^2+^-dependent proteases, in this process. We used high-resolution, dual-color, multiphoton imaging of acute neonatal brain slices expressing a genetically encoded neuronal Ca^2+^ indicator (GCaMP6s), which were treated with propidium iodide (PI), a widely used indicator of cell death (Brana et al., 2002; Buskila et al., 2014), to acquire simultaneously neuronal Ca^2+^ dynamics and a cytotoxicity profile. Our results demonstrate a depth-dependent occurrence of both Ca^2+^ overload and cell death, exacerbated by prolonged incubation. This Ca^2+^ overload resulted in altered physiology, where neurons with Ca^2+^ overload do not respond to stimulus-evoked or seizure-evoked network activity. Using pharmacology, we show that calpain-mediated processes activated during incubation contribute to trauma-induced cell death over time. Thus, our results establish thresholds for imaging noninjured tissue and provide spatiotemporal characteristics and a mechanism of trauma-induced injury in the neocortex of acute neonatal brain slices.

## Materials and Methods

### Study design and animals

All experiments were conducted using a protocol approved by the Institutional Animal Care and Use Committee at The University of Iowa. We used transgenic mice expressing a genetically encoded Ca^2+^ indicator, GCaMP6s, under the *Thy1* promoter [C57BL/6J-Tg (Thy1-GCaMP6s) GP4.3Dkim/J, Jackson Laboratory, strain #024275]. Acute brain slices were prepared at postnatal days 8 to 12 (P8–12) using hemizygous and homozygous, 9 male and 5 female Thy1GCaMP6s mouse pups. We used a total of 27 slices (8 pups) that were incubated in artificial cerebrospinal fluid (aCSF) alone, 16 slices (4 pups) incubated in the calpain inhibitor MDL-28170 (MDL), and 12 slices (3 pups) incubated in the calpain-2-specific inhibitor (Cal 2-1). We used eight staurosporine-treated and five aCSF-treated slices (two pups) to evaluate staurosporine-induced cell death. All the mice were housed in a temperature- and humidity-controlled vivarium with *ad libitum* access to food and water on a 12 h light/dark cycle.

### Preparation of acute brain slices

Mouse pups were anesthetized using isoflurane inhalation and decapitated. Each brain was removed and placed in ice-cold, aCSF containing the following (in mM): 116 NaCl, 3.3 KCl, 1.3 CaCl_2_, 1.25 NaH_2_PO_4_, 25 NaHCO_3_, 10 D-glucose, and 20 D-mannitol along with a high MgCl_2_ concentration (2 mM) and kynurenic acid (2 mM) to block glutamatergic receptors (osmolarity, ∼300 mOsm). The aCSF was saturated with carbogen (95% O_2_ and 5% CO_2_) to maintain a pH of 7.3–7.4. Thick coronal sections (450 µm, commonly used at this age) containing the sensory neocortex were cut using a vibratome (Leica VT1000S, new blade used after every single use) while submerged in ice-cold aCSF, as described previously (Leinekugel et al., 1997; Dzhala et al., 2012; Glykys et al., 2019; Takezawa et al., 2023). We also cut 350 µm slices (common adult thickness) for thickness comparison experiments. The brain slices were placed in an interface holding chamber containing aCSF, devoid of kynurenic acid and with 1.3 mM MgCl_2_, at room temperature for 30 min. Next, the temperature was slowly increased to and maintained at 30°C (recovery time). Per prior protocols, the slices were incubated for at least 1 h after sectioning before being transferred to the recording chamber (Glykys and Mody, 2007; Glykys et al., 2009; Takezawa et al., 2023).

### Pharmacological interventions

See below, under Reagents, for a detailed description of pharmacological agents and other chemicals used in this study. Depending on their solubility, stock solutions were prepared for all compounds in dimethyl sulfoxide (DMSO; Sigma-Aldrich # 472301) or regular aCSF. The stocks were then diluted to desired concentrations in aCSF before perfusion. Whenever appropriate, vehicles contained identical DMSO concentrations to respective stock solutions of the drugs (all ≤0.05%). All solutions (vehicle and drugs) were maintained at room temperature and perfused at ∼2 ml/min through a line heater. The solutions took <30 s to reach the recording chamber. To determine the contribution of calpain-mediated processes in cell death, neonatal slices were either incubated in a nonspecific calpain inhibitor MDL-28170 (30 µM, 1–5 h), calpain-2-specific inhibitor Cal 2-1 (0.5 µM, 2–5 h), or vehicle (1–5 h), following 30 min of initial recovery in regular aCSF. Before multiphoton imaging, individual slices were treated with PI (10 µM for 15–30 min). To estimate staurosporine-induced cell death, all slices were incubated either in staurosporine or vehicle for 4 h in a submerged chamber (5 ml volume) saturated with carbogen before PI treatment and multiphoton imaging. Seizure-like activity was generated in acute brain slices by applying 4-aminopyridine (4-AP) at 100 µM, ∼20 min before starting the recordings (Chesnut and Swann, 1988; Perreault and Avoli, 1991; Langton et al., 2022; Liddiard et al., 2024).

### Focal NMDA puffs

Pulled glass micropipettes (0.5–1 MΩ resistance, tip size 10–20 μm) were filled with 125 µM NMDA and placed on the neocortical surface of acute brain slices. NMDA was applied with a brief puff (3 psi, 100 ms) using a pressure ejector (MPPI-2, Applied Scientific Instruments).

### Multiphoton imaging

Slices were placed in a submerged chamber constantly perfused with recording aCSF bubbled with carbogen and maintained at 30°C. The location of the sensory neocortex was determined using epifluorescence. Two-photon laser scanning microscopy was performed using the Bruker Ultima galvo-resonant system mounted on an Olympus BX51WIF upright microscope with a water immersion objective (20×, 1.0 N.A.). A single Ti:sapphire tunable laser (Mai Tai HPDS; Spectra-Physics) was used for two-photon excitation. Different excitation wavelengths, ranging from 860 to 1,040 nm, were tested for dual-color acquisition of GCaMP6s and PI emissions. An excitation wavelength of 920 nm was selected as it was optimal for Ca^2+^-dependent excitation of GCaMP6s and generated a 50% peak signal of PI (Extended Data Fig. 1-1*A*). Scanning was performed with galvo-mirrors. Emitted light was bandpass filtered at 565 nm using a dichroic mirror (T510lpxrxt, Chroma), and green and red emission wavelengths were isolated using specific filters: 525/35 nm (green) and 595/25 nm (red). Two GaAsP or multi-alkali photomultiplier tubes (PMT, Hamamatsu Photonics) were used to simultaneously acquire green and red signals. Three-dimensional stacks (3D) of raster scans were acquired at a 512 × 512 pixel resolution, beginning at the top of the slices to a depth of 300 µM (step size, 2–3 μm). The acquired images contain multiple layers of the sensory neocortex. All images were acquired at 1× digital zoom. The laser power was linearly increased throughout the stack acquisition (16–109 mW from top to bottom) to offset the scattering/absorption of photons in deeper tissue.

### Image processing and segmentation

The 3D stacks acquired using two-photon laser scanning microscopy were split into two channels (green and red), converted to 8 bit TIFF format, and processed separately. The *Z*-stacks were background subtracted and smoothened (median filter; radius, 2), and maximum intensity projections (MIPs) were generated for every five images, each representing a 10–15 μm deep section of brain tissue. To detect GCaMP-filled and PI^+^ cells, a threshold of five times the background's standard deviation (SD) was used to apply a binary mask over MIPs acquired from the green and red channels, respectively. GCaMP-filled and PI^+^ cells were then segmented using the “analyze particles” function (ImageJ) using specific filters (size for PI^+^ cells, 10–100 μm; size for GCaMP-filled cells, 20–200 μm; circularity, 0.5–1 for both). To eliminate false PI^+^ cell detections due to the green bleed-through signal, a subset of PI^+^ cells were manually detected (*n* = 284 cells), and red/green ratios for each detection were calculated. A Gaussian function was fitted to their frequency distribution to obtain the mean and SD (0.3 ± 0.7). A threshold for red/green intensity ratios obtained from the test dataset (mean minus 1 SD: −0.4) was used to eliminate false red detections from the experimental dataset acquired with automated selection (*n* = 4,087 red/green ratios; Extended Data Fig. 1-1*B*). A custom-built macro automatically updated the ROIs representing GCaMP-filled/PI^+^ detections to their corresponding *z*-planes. To determine GCaMP-filled/PI^+^ colocalization, the centroids of the ROIs detected using the “red” and “green” stacks were matched using a custom-made macro in Igor Pro (WaveMetrics).

To measure the neuronal Ca^2+^ transients, raw images in time series were background subtracted, smoothened (median filter; radius, 2), and compressed into MIPs. Circular ROIs representing neuronal somas and concentric, doughnut-shaped ROIs representing the neuropil were generated on the MIPs using a semiautomated, custom-built macro. The neuropil fluorescence was subtracted from the corresponding somatic fluorescence for each frame. To determine the characteristics of evoked Ca^2+^ responses, we performed baseline subtraction and normalization (Δ*F* / *F*_0_) of the neuropil-subtracted Ca^2+^ signal for each neuron using the following equation: (*F_t_* − *F*_0_) / *F*_0_, where *F_t_* is the fluorescence intensity of a given frame and *F*_0_ is the average baseline fluorescence.

### Reagents

All aCSF solutes, kynurenic acid, PI, 4-AP, and MDL-28170 (MDL) were obtained from Sigma-Aldrich. Staurosporine was obtained from Tocris. Calpain-2-specific inhibitor Calpain 2-IN-1 (Cal 2-1) was obtained from MedChemExpress. PI was diluted from an aCSF stock solution (1 mg/ml, 1,000×) to a final 1 μg/ml concentration. MDL was diluted from a DMSO stock solution (60 mM, 2,000×) to a final concentration of 30 μM. 4-AP was diluted from a stock prepared in water (100 mM, 1,000×) to a final concentration of 100 μM. Cal 2-1 was diluted from DMSO stock solution (5 mM, 10,000×) to a final concentration of 0.5 μM. Staurosporine (100 μg) was dissolved in DMSO to make a 5 mM stock (42.9 μl), which was then dissolved in 10 ml aCSF to obtain a final concentration of 20 μM. All stock solutions were stored at −20°C and used within 3 months of preparation.

### Statistics

The total number of (or % normalized) PI^+^ and GCaMP-filled cells were calculated at different depths (from 0 to 300, 30 μm steps containing 2–3 MIPs) for multiple slices and plotted as a normalized fraction or total number across a bin of 30 μm. Exponential decays were fitted to the % normalized cells to obtain decay constants (*τ*). To measure neuropil GCaMP6s intensity across depth, median fluorescence intensities for each 30 μm step were plotted. To determine the effect of incubation time, the number or % normalized cells versus depth plots were generated for slices grouped according to a range of incubation durations (0–1, 1–2, 2–3, 3–5 h) before fitting the exponential decay functions. The normality of distributions was determined using the Shapiro–Wilk test and QQ plots. Statistical tests for parametric data were used for normal distributions, while lognormal distributions were log-transformed before applying parametric tests. Normally distributed data are presented as mean ± 95% confidence interval (CI). Nonparametric data are presented as median ± interquartile range (IQR). Data representing multiple depths within the same perturbation were analyzed using repeated-measures one-way ANOVA with Dunnett's test for multiple comparisons. Unpaired parametric data across various time points were analyzed using one-way ANOVA. Data representing multiple depths were compared across groups (or perturbations) using two-way ANOVA with Tukey's or Sidak's post hoc tests, depending on the number of multiple comparisons. Data points representing individual slices or cells were compared across groups using a two-tailed, unpaired *t* test (parametric data) or Mann–Whitney *U* test (nonparametric data). For statistical correlations, data were normalized to slice total (PI^+^ and GCaMP-filled cells) or slice maximum value (median neuropil GCaMP6s intensities). To determine a correlation between median neuropil intensities obtained from XY grids and PI^+^ cells, the grids with zero PI^+^ cells were eliminated from the analysis. Spearman's correlation (nonparametric data) was used to identify a statistically significant correlation. Statistical significance was considered at *p* < 0.05. For detailed statistics, see Table 1.

**Table 1.** Statistical table

Figure	Structure	Type of test	Results
Fig. 1*C*	Normal	RM one-way ANOVA	PI^+^: *F*_(2,52.2) _= 42.7, *p* < 0.0001
			GCaMP-filled: *F*_(2.58,67) _= 18.8, *p* < 0.0001
Fig. 1*D* (inset)	Normal	Unpaired *t* test (two-tailed)	*t* = 0.037, df = 32, *p* = 0.97
Fig. 1*F*	Nonparametric	Spearman's correlation	Spearman's *ρ* = 0.66, *p* < 0.0001
Extended Data Fig. 1-2*C*	Normal	Two-way RM ANOVA	Interaction: *F*_(9,99) _= 4.025, *p* = 0.0002
			Depth: *F*_(9,99) _= 16.8, *p* < 0.0001
			Treatment: *F*_(1,11) _= 12.3, *p* = 0.0049
Extended Data Fig. 1-2*D*	Normal	Unpaired *t* test (two-tailed)	*t* = 3.51, df = 11, *p* = 0.005
Extended Data Fig. 1-2*E*	Normal	Unpaired *t* test (two-tailed)	*t* = 2.76, df = 11, *p* = 0.02
Extended Data Fig. 1-2*F*	Normal	Two-way RM ANOVA	Interaction: *F*_(9,99) _= 4.15, *p* = 0.0001
			Depth: *F*_(9,99) _= 6.18, *p* < 0.0001
			Treatment: *F*_(1,11) _= 12.3, *p* = 0.062
Extended Data Fig. 1-2*G*	Normal	Two-way RM ANOVA	Interaction: *F*_(9,90) _= 2.18, *p* = 0.034
			Depth: *F*_(9,90) _= 14.1, *p* < 0.0001
			Treatment: *F*_(1,10) _= 0.041, *p* = 0.93
Extended Data Fig. 1-3*C*	Normal	Two-way ANOVA	Interaction: *F*_(6,87) _= 0.3, *p* = 0.936
			Time point: *F*_(3,87) _= 9.2, *p* < 0.0001
			Layer: *F*_(2,87) _= 2.42, *p* = 0.095
Extended Data Fig. 1-3*D*	Normal	Two-way ANOVA	Interaction: *F*_(6,82) _= 0.06, *p* = 0.99
			Time point: *F*_(3,82) _= 0.95, *p* = 0.42
			Layer: *F*_(2,82) _= 0.78, *p* = 0.45
Extended Data Fig. 1-4*A*	Normal	Two-way RM ANOVA	Interaction: *F*_(9,351) _= 2.04, *p* = 0.034
			Depth: *F*_(9,351) _= 129.4, *p* < 0.0001
			Slice thickness: *F*_(1,39) _= 1.74, *p* = 0.125
Extended Data Fig. 1-4*B*	Normal	Two-way RM ANOVA	Interaction: *F*_(9,342) _= 2.04, *p* = 0.0007
			Depth: *F*_(9,342) _= 52.51, *p* < 0.0001
			Slice thickness: *F*_(1,38) _= 1.81, *p* = 0.186
Extended Data Fig. 1-4*C*	Normal	Two-way RM ANOVA	Interaction: *F*_(9,216) _= 0.08, *p* = 0.99
			Depth: *F*_(9,216) _= 91.3, *p* < 0.0001
			Age: *F*_(1,24) _= 0.36, *p* = 0.56
Extended Data Fig. 1-4*D*	Normal	Two-way RM ANOVA	Interaction: *F*_(9,306) _= 0.87, *p* = 0.56
			Depth: *F*_(9,306) _= 54.16, *p* < 0.0001
			Age: *F*_(1,34) _= 10.49, *p* = 0.0027
Fig. 2*B*	Normal	RM one-way ANOVA	PI^+^: *F*_(2,52.2) _= 42.7, *p* < 0.0001
			Neuropil: *F*_(2,52.2) _= 42.7, *p* < 0.0001
Fig. 2*C*	Nonparametric	Spearman's correlation	Spearman's *ρ* =0.68, *p* < 0.0001
Fig. 2*E*	Normal	Pearson's correlation	Pearson's *r* = 0.61, *p* < 0.0001
Fig. 3*B*	Normal	Two-way RM ANOVA	Interaction: *F*_(27,198) _= 1.578, *p* = 0.043
			Depth: *F*_(9,198) _= 107.1, *p* < 0.0001
			Treatment: *F*_(3,22) _= 2.75, *p* = 0.07
Fig. 3*E*	Normal	One-way ANOVA	PI^+^: *F*_(3,22) _= 3.06, *p* = 0.049
			Dunnett's multiple-comparisons test
			0–1 vs 1–2: *p* = 0.91, 0–1 vs 2–3: *p* = 0.99
			vs 3–5: *p* = 0.033
			GCaMP-filled: *F*_(3,18) _= 1.7, *p* = 0.2
			Dunnett's multiple-comparisons test
			0–1 vs 1–2: *p* = 0.97, 0–1 vs 2–3:
			*p* = 0.96
			0–1 vs 3–5: *p* = 0.13
Fig. 5*C*	Nonparametric	Wilcoxon matched pairs test	*W* = 21, *p* = 0.031
Fig. 5*D*	Nonparametric	Wilcoxon matched pairs test	*W* = 21, *p* = 0.031
Fig. 5*F*	Nonparametric	Wilcoxon matched pairs test	*W* = 21, *p* = 0.031
Fig. 5*F*	Nonparametric	Wilcoxon matched pairs test	*W* = 8, *p* = 0.375
Fig. 6*B*	Normal	Two-way RM ANOVA	Interaction: *F*_(9,369) _= 3.32, *p* = 0.0006
			Depth: *F*_(9,369) _= 48.1, *p* < 0.0001
			Treatment: *F*_(1,41) _= 8.51, *p* = 0.0057
Fig. 6*B* (inset)	Normal	Unpaired *t* test (two-tailed)	*t* = 3.19, df = 41, *p* = 0.002
Fig. 6*D*	Normal	Two-way ANOVA	Interaction: *F*_(3,34) _= 0.97, *p* = 0.41
			Depth: *F*_(3,34) _= 4.57, *p* = 0.0091
			Treatment: *F*_(1,34) _= 15.4, *p* = 0.0004
			Sidak's multiple-comparisons test
			<1 h: *p* = 0.67, 1–2 h: *p* = 0.21, 2–3 h: *p* = 0.023, 3–5 h: *p* = 0.011
Fig. 6*E*	Normal	Two-way ANOVA	Interaction: *F*_(3,33) _= 1.4, *p* = 0.26
			Depth: *F*_(3,33) _= 0.71, *p* = 0.55
			Treatment: *F*_(1,33) _= 14.8, *p* = 0.0005
			Sidak's multiple-comparisons test
			<1 h: *p* = 0.61, 1–2 h: *p* = 0.75, 2–3 h: *p* = 0.64, 3–5 h: *p* = 0.001
Fig. 6*F*	Normal	Two-way RM ANOVA	Interaction: *F*_(9,369) _= 2.17, *p* = 0.023
			Depth: *F*_(9,369) _= 9.09, *p* < 0.0001
			Treatment: *F*_(1,41)_ = 4.19, *p* = 0.047
Fig. 6*F* (inset)	Nonparametric	Mann–Whitney test	*U* = 86, *p* = 0.002
Fig. 6*G*	Normal	Two-way RM ANOVA	Interaction: *F*_(9,369) _= 1.46, *p* = 0.038
			Depth: *F*_(9,369) _= 23.7, *p* < 0.0001
			Treatment: *F*_(1, 41) _= 2.8, *p* = 0.01
Fig. 6*G* (inset)	Normal	Unpaired *t* test (two-tailed)	*t* = 2.15, df = 41, *p* = 0.038
Extended Data Fig. 6-1*B* (right)	Lognormal	One-way ANOVA (Tukey's post hoc test)	ANOVA, *F*_(2,30)_ = 4.65. *p* = 0.017
			Tukey's multiple-comparisons test
			aCSF vs MDL, *p* = 0.033
			aCSF vs Cal 2-1, *p* = 0.046
			MDL vs Cal 2-1, *p* = 0.910
Extended Data Fig. 6-1*B* (right)	Lognormal	One-way ANOVA (Tukey's post hoc test)	ANOVA, *F*_(2,31)_ = 5.79. *p* = 0.007
			Tukey's multiple-comparisons test
			aCSF vs MDL, *p* = 0.005
			aCSF vs Cal 2-1, *p* = 0.188
			MDL vs Cal 2-1, *p* = 0.179

## Results

### Evidence of abundant cell death and neuronal Ca^2+^ overload on the surface of acute brain slices decreasing exponentially with depth

**Figure 1.**
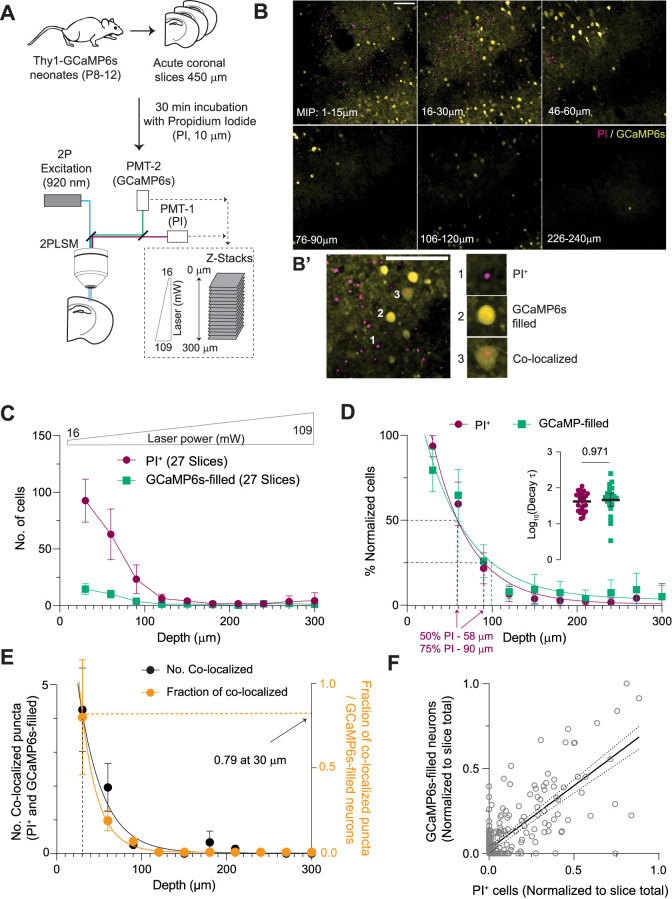
Depth-dependent co-occurrence of PI^+^ cells and GCaMP-filled neurons in acute neonatal brain slices. ***A***, Experimental design. ***B***, Representative MIPs (15 µm depth) of images acquired at different depths. ***B*’**, Example of a PI^+^ cell, a GCaMP-filled neuron, and colocalized puncta. False-positive detections were eliminated using a ratiometric approach (Extended Data Fig. 1-1). PI labeling due to cytotoxic injury was validated by staurosporine-induced apoptosis in acute brain slices (Extended Data Fig. 1-2). ***C***, Reduction in the number of PI^+^ cells and GCaMP-filled neurons with depth in acute brain slices (RM one-way ANOVAs, PI^+^: *F*_(2,52.2)_ = 42.7, *p* < 0.0001, GCaMP-filled: *F*_(2.58,67)_ = 18.8, *p* < 0.0001). ***D***, Normalized fraction of total PI^+^ cells across different depths. Single exponential decay fits show a similar depth-dependent decrease in PI^+^ cells and GCaMP-filled neurons (*τ*_PI+_: 41 ± 10 µm, *R*^2^: 0.75, *τ*_GCaMP-filled_: 65 ± 25 µm, *R*^2^: 0.54; inset, unpaired *t* test). ***E***, Reduction in colocalized puncta (PI^+^ and GCaMP-filled) with depth (*R*^2^: 0.63, *τ*: 30 µm). Right axis, fractional plot (*R*^2^: 0.58, *τ*: 21 µm, 79% in the first 30 µm). ***F***, Significant correlation between PI^+^ cells and GCaMP-filled neurons, identified from individual MIPs, normalized to slice totals (Spearman's *ρ* = 0.66, *p* < 0.0001, linear regression slope: 0.75, *R*^2^ = 0.6, *p* < 0.0001). PI^+^ cells and GCaMP-filled neurons show no cortical layer-specific localization (Extended Data Fig. 1-3) or postnatal age-dependent distribution change across depth (Extended Data Fig. 1-4). However, their distribution across depth depends on the acute slice thickness (Extended Data Fig. 1-4). Mean ± 95% CI. Scale bar, 100 µm. See also Movies 1 and 2.

First, we evaluated the anatomical localization of trauma- and incubation-induced cell death and Ca^2+^ overload (GCaMP-filled neurons) in the neocortex of neonatal brain slices. We acquired high-resolution, dual-color multiphoton 3D scans (0–300 µm; step size, 2–3 µm) of the somatosensory region from acute Thy1-GCaMP6s slices (Chen et al., 2012) labeled with PI (Buskila et al., 2014), a known marker for cell death (Fig. 1*A*). We then obtained MIPs for every 10–15 µm step from the 3D stacks (Fig. 1*B*). These MIPs, representing progressive depths, were used to detect GCaMP-filled neurons and PI^+^ cells. False-positive detections of the PI signal were eliminated using a ratiometric and threshold-based approach (Materials and Methods; Extended Data Fig. 1-1). The numbers of PI^+^ cells and GCaMP-filled neurons were highest in the superficial layers (0–30 µm) and decreased with increasing depth (Fig. 1*C*). To determine the precise decay rates across depth, we fitted single exponentials to the percent normalized PI^+^ and GCaMP-filled cell counts (Fig. 1*D*). Both had a similar rate of decay (*τ*_PI_^+^, 41 ± 10 µm; *τ*_GCaMP-filled_, 65 ± 25 µm) with ∼75% of average PI^+^ and GCaMP-filled cells in the top 0–90 and 0–100 µm of the slice, respectively. We also found fewer colocalized puncta (GCaMP-filled and PI^+^ neurons) with increasing depth (Fig. 1*E*; ∼79% colocalized neurons were in the uppermost 30 µm). There was also a significant correlation between cell death (PI^+^) and GCaMP-filled neurons across tissue depths (Fig. 1*F*). Thus, these data indicate a depth-dependent co-occurrence of cell death and elevated neuronal Ca^2+^.

10.1523/ENEURO.0007-24.2024.f1-1 Fig 1-1 **Eliminating false PI^+^ cell detections by emission ratio analysis. A)** *Left:* MIPs showing excitation of PI and GCaMP6  s using multiple 2P wavelengths. *Right*: Excitation wavelength vs. % emission intensity for PI and GCaMP6  s. There was ∼54% PI emission intensity at 920  nm, a wavelength widely used for Ca^2+^-dependent GCaMP6  s excitation (n = 8 slices). **B)** *Left:* To eliminate false PI ^+ ^-cell detections due to a green bleed-through signal, a threshold was identified (-0.4, magenta dashed line) from plotting the distribution of Red/Green ratios from a dataset of manually selected “True-positive PI^+^ cells.” *Right:* Lognormal distribution of Red/Green ratios of automated PI^+^ detections across all slices. Detections below the threshold (dashed line) were eliminated as false positives (1,543 cells eliminated). Data represented as mean ± 95% CI. Scale bar = 100  µm. Download Fig 1-1, TIF file.

**Figure 2.**
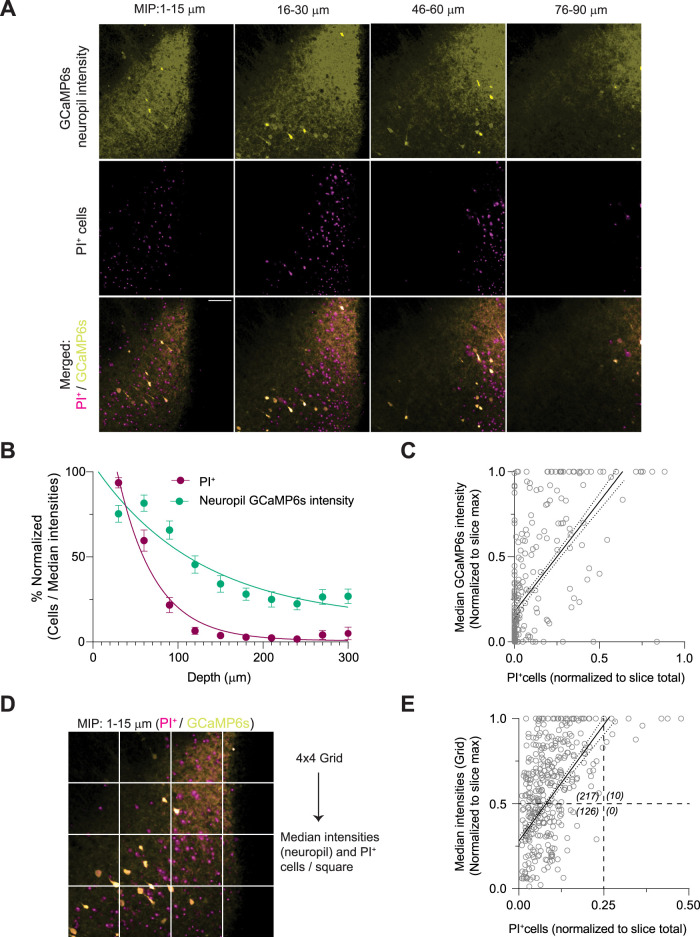
Spatial correlation between PI^+^ cells and the neuropil Ca^2+^ signal. ***A***, Representative MIPs showing PI^+^ cells and neuropil GCaMP6s signal in an acute neonatal brain slice across different depths. ***B***, Depth-dependent decrease in the median GCaMP6s intensities within the neuropil (RM one-way ANOVA, *F*_(2,52.2)_ = 42.7, *p* < 0.0001, *τ*_neuropil_: 131 ± 57 µm, *R*^2^: 0.45). PI^+^ data and fit from Figure 1*D* are displayed for comparison. ***C***, Number of PI^+^ cells and median neuropil GCaMP intensities of individual MIPs (both normalized) showed a significant correlation (Spearman's *ρ* = 0.68, *p* < 0.0001, linear regression slope: 1.3, *R*^2^ = 0.4, *p* < 0.0001). ***D***, Placement of identical regions of interest (ROIs) in a 4 × 4 grid over a MIP. ***E***, The number of PI^+^ cells and median neuropil GCaMP intensities of individual grids (normalized) showed a significant correlation (Pearson's *r* = 0.61, *p* < 0.0001, linear regression slope: 2.7, *R*^2^ = 0.4, *p* < 0.0001). Numbers in parentheses represent data points in each quadrant. Mean ± 95% CI. Scale bar, 100 µm.

10.1523/ENEURO.0007-24.2024.f1-2 Fig 1-2 **Validation of PI ^+ ^-cell detection across various depths in acute neonatal brain slices. A)** Experimental design. **B**) MIPs representing different depths, acquired from brain slices (post-natal days 8-12), incubated for 4 hours in aCSF alone (*top*) and staurosporine (20  µM*, bottom*). **C)** Staurosporine significantly increased the number of PI ^+ ^-cells in acute neonatal brain slices across various depths (Two-way ANOVA, interaction: F(9, 99) = 4, p = 0.0002, treatment: F(1, 11) = 12.3, p = 0.0049). **D)** Increase in the total number of PI ^+ ^-cells (unpaired t-test, p = 0.005*)* and (**E**) in decay constants with staurosporine pre-treatment (unpaired t-test, p = 0.02) calculated from individual slices. **F)** Increase in colocalized puncta across tissue depth with staurosporine pre-treatment (Two-way ANOVA, interaction: F(9, 99) = 4.15, p < 0.0001, treatment: F(1, 11) = 12.3, p = 0.062). **G)** Although we found a modestly significant interaction between control and staurosporine-treated slices, post-hoc multiple comparisons confirmed no significant increase in GCaMP6-filled neurons due to staurosporine treatment (Two-way ANOVA, interactions: F(9, 90) = 2.18, p < 0.034, Treatment: F(1, 10) = 0.041, p = 0.93, Sidak’s test for multiple comparisons p > 0.05). aCSF: 5 slices; staurosporine: 8 slices. Mean ± 95% CI. Scale bar = 100  µm. Download Fig 1-2, TIF file.

**Figure 3.**
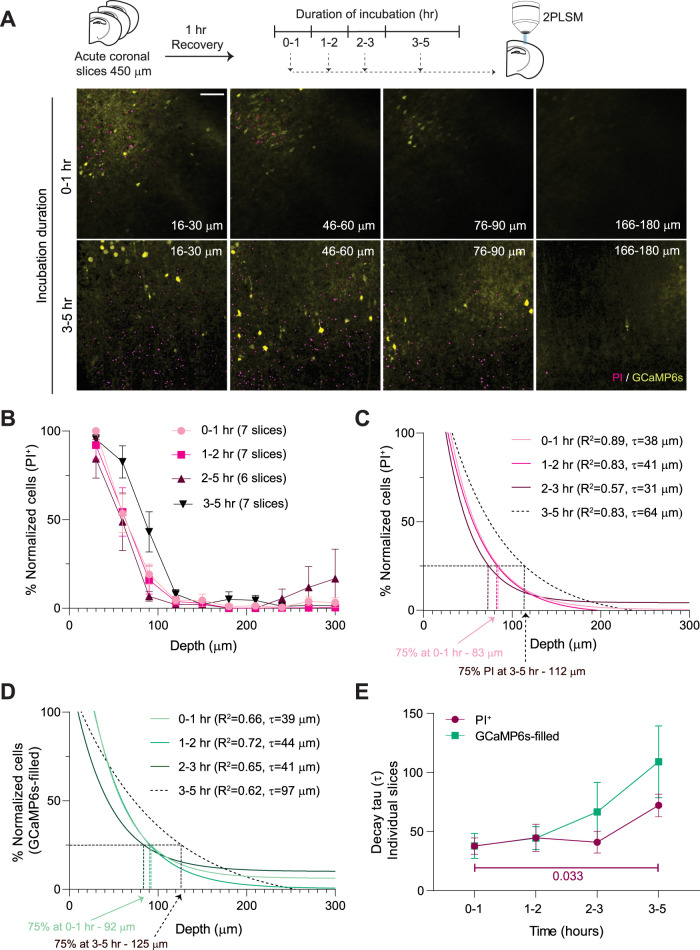
Prolonged incubation times increase the number of PI^+^ cells and GCaMP-filled neurons in deeper tissue. ***A***, Top, Experimental design. Bottom, MIPs representing different depths acquired from slices after 0–1 and 3–5 h incubations. ***B***, Normalized number of PI^+^ cells across depth at different incubation times (two-way RM ANOVA, interaction: *F*_(27,198)_ = 1.58, *p* = 0.043; treatment: *F*_(3,22)_ = 2.75, *p* = 0.07). ***C***, Increase in the percentage of PI^+^ cells with increasing incubation times. *R*^2^ and *τ* from a single exponential fit to the means. ***D***, Similarly, a slower exponential decay of CaMP6s-filled neurons was observed with more extended incubation (*R*^2^ and *τ* of exponential decay fit to the means). ***E***, Increase in the decay constants measured from individual slices (% PI^+^ cells) during prolonged incubation (one-way ANOVA, *F*_(3,22)_ = 3.06, *p* = 0.049, Dunnett's test, 0–1 vs 3–5 h: *p* = 0.033). Although not significant, the decay constants for the GCaMP-filled neurons also showed a trend toward an increase with prolonged incubations (one-way ANOVA, *F*_(3,18)_ = 1.7, *p* = 0.2). Mean ± 95% CI. Scale bar, 100 µm.

10.1523/ENEURO.0007-24.2024.f1-3 Fig 1-3 **Layer-specific distribution of PI^+^ and GCaMP-filled neurons A)** Experimental design describing a strategy for imaging superficial and deeper layers of the primary somatosensory cortex. **B)** Representative image showing PI^+^ and GCaMP-filled distribution neurons across superficial and deeper layers. **C)** Density of PI^+^ cells and **D)** GCaMP-filled neurons in superficial and deeper layers, measured from acute brain slices at different durations of incubation, showed no significant difference between layers (PI^+^: Two-way ANOVA, interaction: F(6, 87) = 0.3, p = 0.936; GCaMP-filled: Two-way ANOVA, interaction: F(6, 82) = 0.06, p = 0.99). Data represented as mean ± 95% CI. Scale bar = 100  µm. Download Fig 1-3, TIF file.

**Figure 4.**
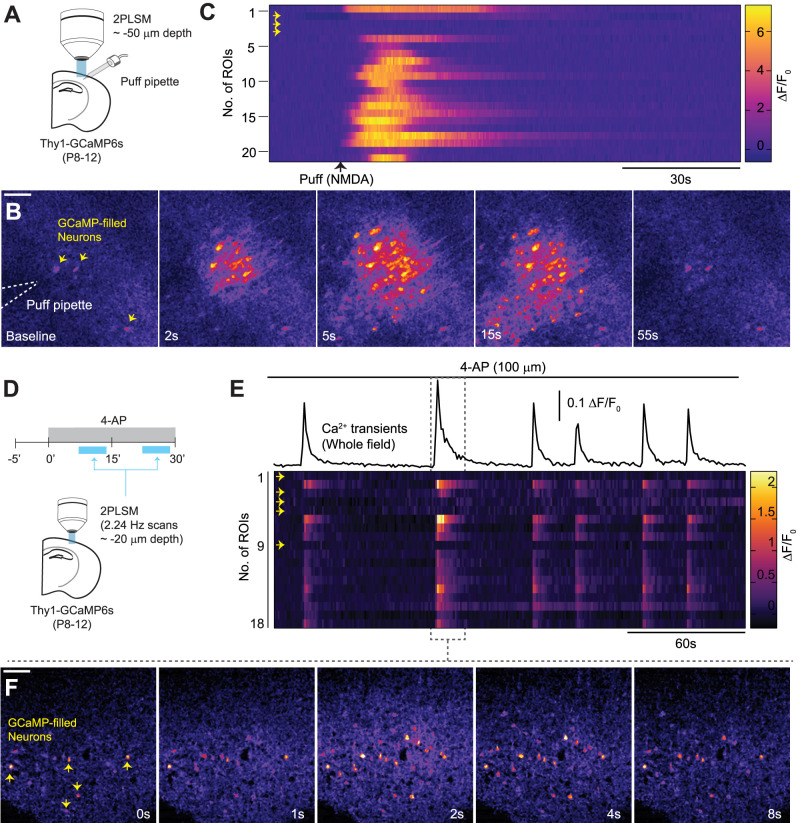
Activity-dependent Ca^2+^ dynamics in healthy and GCaMP-filled neurons. ***A***, Experimental design showing placement of a puff pipette on an acute slice. ***B***, Representative images of propagating Ca^2+^ transient evoked by a brief NMDA puff (125 µM, 100 ms). GCaMP-filled neurons with tonically high baseline Ca^2+^ signals are indicated by arrows (yellow). ***C***, Heatmap shows Ca^2+^ transients evoked in multiple healthy neurons, while GCaMP-filled neurons (yellow arrows) showed no evoked Ca^2+^ activity (*n* = 12 GCaMP-filled neurons in 3 slices). See Movie 1. ***D***, Experimental design of multiphoton acquisition of 4-AP-induced Ca^2+^ transients in acute slices. ***E***, Top, Representative trace showing 4-AP-induced whole-field Ca^2+^ activity in an acute brain slice. Bottom, Heatmap shows 4-AP-induced Ca^2+^ transients acquired from multiple healthy and GCaMP-filled neurons (yellow arrows). Note that GCaMP-filled neurons showed no Ca^2+^ transients (*n* = 27 GCaMP-filled neurons in 4 slices). ***F***, Representative images showing spatiotemporal propagation of a single 4-AP-induced Ca^2+^ transient. See Movie 2. Scale bar, 100 µm.

10.1523/ENEURO.0007-24.2024.f1-4 Fig 1-4 **Effects of slice thickness and postnatal age on inherent injury in acute brain slices A)** Differences in normalized PI^+^ cells across depths between 350 and 450  µm thick acute brain slices (Two-way ANOVA, Interaction: F(9, 351) = 2.04, p = 0.034), with an increase at 300  µm depth in the 350  µm thick slices (Sidak’s multiple comparisons, p = 0.038). **B)** Significant difference between the distribution of GCaMP-filled neurons across depths (Two-way ANOVA, Interaction: F(9, 342) = 2.04, p = 0.0007), with an increase at 300  µm in the 350  µm thick slices (Sidak’s multiple comparisons, p = 0.0001). No postnatal age difference in **C)** PI^+^ cells (Two-way ANOVA, Interaction: F(9, 216) = 0.08, p = 0.99) or **D)** GCaMP-filled neurons (Two-way ANOVA, Interaction: F(9, 306) = 0.87, p = 0.56). Data represented as mean ± 95% CI. Download Fig 1-4, TIF file.

To confirm the efficacy of PI labeling and the segmentation of PI^+^ cells, we induced apoptosis in acute neonatal brain slices by treating them with staurosporine (20 µM for 4 h; Hanse and Gustafsson, 1994; Zhou et al., 2001). We acquired dual-color images from staurosporine-treated and aCSF-treated (control) brain slices. PI^+^ cells and GCaMP-filled neurons were present at different depths across the slice (Extended Data Fig. 1-2*A*,*B*). Staurosporine significantly increased the number of PI^+^ cells at various depths compared with aCSF, with a significant increase in decay constants and total PI^+^ cells per slice (Extended Data Fig. 1-2*C–E*). Staurosporine also significantly increased colocalized puncta across different tissue depths (Extended Data Fig. 1-2*F*). These results validate our PI labeling and cell segmentation approach.

Next, we tested if PI^+^ cells and GCaMP-filled neurons were localized to specific cortical layers. Although the density of PI^+^ cells and GCaMP-filled neurons was slightly higher in deeper layers, the difference between deeper and superficial layers was not statistically significant (Extended Data Fig. 1-3). We then tested if slice thickness and postnatal age subdistribution altered trauma-related cell death and Ca^2+^ overload in acute brain slices. We found that thinner slices (350 µm, common thickness used in adult brain slices) had a significant increase in PI^+^ cells and GCaMP-filled neurons at a depth of 270–300 µm, near the opposite surface of the slice (Extended Data Fig. 1-4*A*,*B*). We did not observe a postnatal age-dependent effect (P8–9 vs P10–12) on the distribution of PI^+^ cells and GCaMP6-filled neurons across depth (Extended Data Fig. 1-4*C*,*D*).

### Cell death and elevated neuropil Ca^2+^ signal are correlated

Elevated Ca^2+^ signal in the neuropil is often associated with injury (Berridge et al., 2003). We examined the relationship between cell death and neuropil Ca^2+^ signal across different depths in acute neonatal brain slices (Fig. 2*A*). Like PI^+^ cells, the normalized median neuropil intensities displayed an exponential decrease with depth (Fig. 2*B*). The normalized number of PI^+^ cells and median neuropil GCaMP intensities acquired from individual MIPs showed a significant correlation (Fig. 2*C*). Next, we evaluated the correlation between neuropil GCaMP intensities and the number of PI^+^ cells in 2D space (*x*–*y* dimensions). We found that PI^+^ cells and median neuropil Ca^2+^ signals, measured from individual ROIs within a 4 × 4 grid, showed a significant correlation (Fig. 2*E*), indicating that PI^+^ cells segregate with elevated Ca^2+^ in the neuropil.

### Prolonged incubation of acute neonatal brain slices exacerbates cell death and neuronal Ca^2+^ overload

Along with the trauma-induced injury during slice preparation, longer incubations of acute brain slices can also adversely affect cell viability (Fukuda et al., 1995). To determine the dynamic changes in trauma-induced injury with prolonged incubations, we compared the percentage change of PI^+^ cells and GCaMP-filled neurons across depth following prolonged incubation times (Fig. 3*A*). Although the distribution of PI^+^ cells across tissue depth was similar for all incubation times of 3 h or less, prolonged (3–5 h) incubations caused a significant increase in PI^+^ cells (Fig. 3*B*), reflected in slower exponential decays (Fig. 3*C*). Although the distribution of PI^+^ cells across tissue depth was similar for all incubation times of 3 h or less, prolonged (3–5 h) incubations caused a significant increase in the number of PI^+^ cells (Fig. 3*B*), reflected in slower exponential decays (Fig. 3*C*). Notably, following 0–1 h incubation, 75% of PI^+^ cells were localized in the upper 83 µm of tissue, which increased to the upper 112 µm of tissue following 3–5 h incubation (Fig. 3*C*). We found a similar increase in the exponential decay constants of GCaMP-filled neurons following 3–5 h of incubations (Fig. 3*D*). When averaged from individual slices, the decay constants increased with longer incubation times for PI^+^ cells and GCaMP-filled neurons but did not reach statistical significance for the latter (Fig. 3*E*). These results show that incubation durations above 3 h adversely affect cell viability, increasing neuronal Ca^2+^ load and cell death in the most superficial layers.

### Altered responses of GCaMP-filled neurons during evoked and seizure-induced activity

During slice preparation, traumatic injury to neurons and their processes can significantly alter neuronal and synaptic physiology (Kirov et al., 1999; Dzhala et al., 2012). Therefore, we evaluated the response of GCaMP-filled neurons during induced network activity. These neurons were characterized by tonically high fluorescence and a lack of Ca^2+^ transients during external stimuli (125 µM NMDA puff, 100 ms) or during seizure-like activity induced by 100 µM of 4-AP (Fig. 4; Movies 1, 2). None of the identified filled neurons responded to NMDA puffs or to 4-AP seizure evoked activity (total 39 neurons from 7 slices).

**Figure 5.**
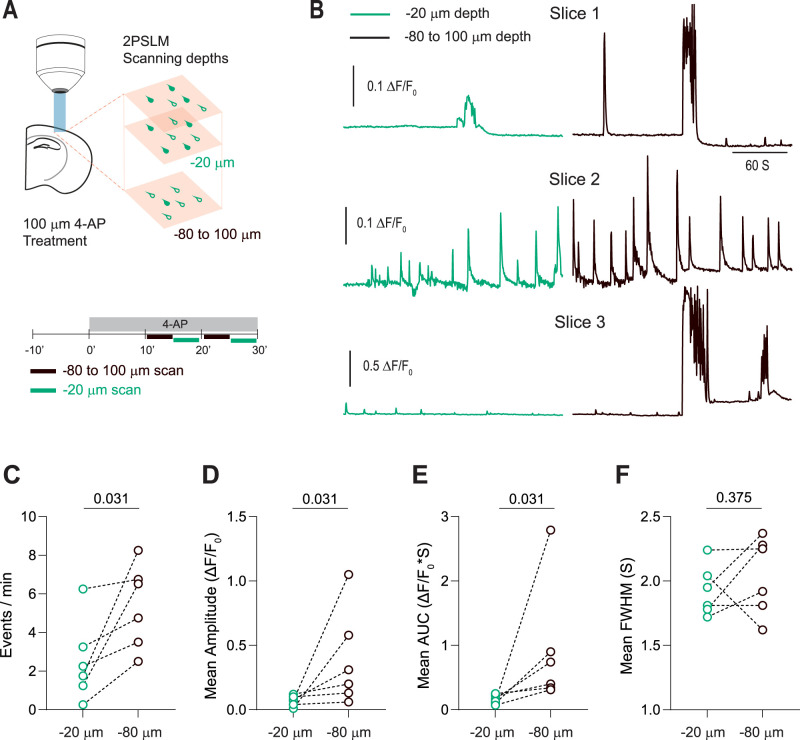
Depth-dependent alterations in seizure-induced neuronal Ca^2+^ transients. ***A***, Schematic depicting experimental design. ***B***, Representative traces showing synchronous, whole-field Ca^2+^ transients obtained at different depths. See Movie 3. Synchronous neuronal Ca^2+^ activity in deeper tissue showed a significant increase in (***C***) Ca^2+^ transient frequency, (***D***) mean amplitude, and (***E***) mean area under the curve (AUC), without a significant change in the Ca^2+^ transient duration measured as full-width at half-maximum (***F***, FWHM). Wilcoxon matched pairs test, *n* = 6 slices.

Next, since the superficial tissue is more susceptible to traumatic injury during slice preparation, we tested if seizure-related Ca^2+^ dynamics in superficial neurons were altered compared to neurons in deeper tissue. Seizure-like activity was induced by applying 4-AP (100 µM), and neuronal Ca^2+^ transients were imaged at different depths (20 or 80–100 µm from the surface; Fig. 5*A*). 4-AP application induced synchronous, transient neuronal Ca^2+^ activity in the superficial and deep tissue (Fig. 5*B*, Movie 3). However, the synchronous neuronal Ca^2+^ transients had significantly higher frequencies, amplitudes, and areas under curve in the deeper tissue than the superficial ones (Fig. 5*C–F*). These results show that GCaMP-filled neurons have altered physiology and modified the network activity.

### Calpain inhibition during acute brain slice incubation mitigates cell death

**Figure 6.**
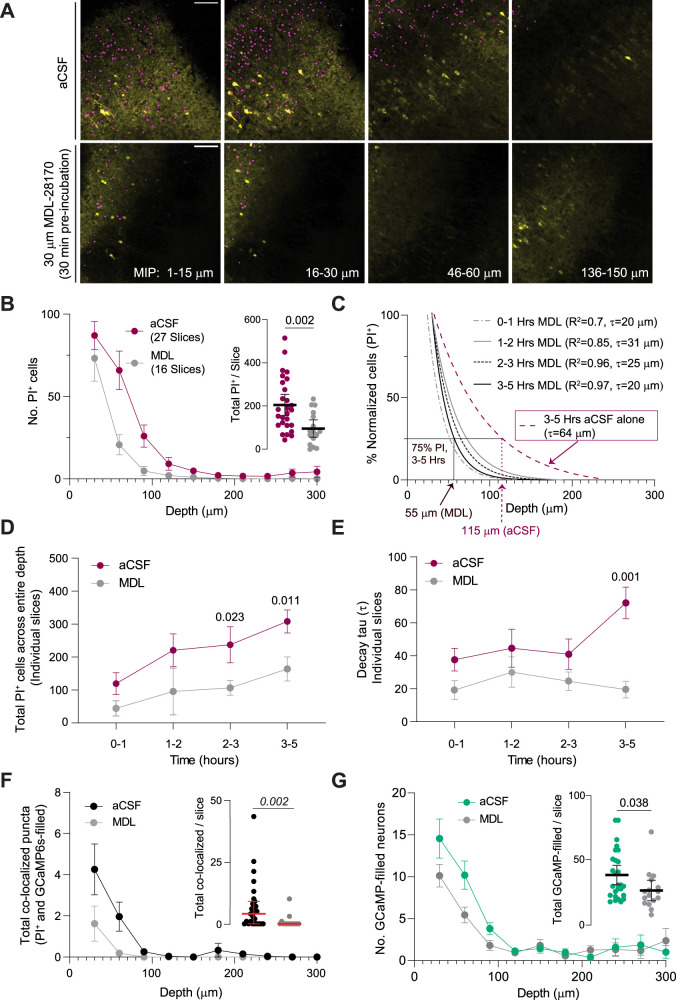
Inhibiting calpains during incubation mitigates cell death in acute brain slices. ***A***, MIPs at different depths from brain slices incubated in aCSF alone (top) or with a calpain inhibitor (MDL, bottom). ***B***, MDL significantly reduced the number of PI^+^ cells across different depths (two-way ANOVA, interaction: *F*_(9,369)_ = 3.32, *p* = 0.0006; treatment: *F*_(1,41)_ = 8.51, *p* = 0.0057) and the total PI^+^ cells per slice (inset, unpaired *t* test). ***C***, Faster decays in the percent of PI^+^ cells with MDL incubation. *R*^2^ and *τ* of single exponential fits to the means. ***D***, ***E***, MDL-28170 significantly decreased the total number of PI^+^ cells (two-way ANOVA, interaction: *F*_(3,34)_ = 0.97, *p* = 0.41; treatment: *F*_(1,33)_ = 15.4, *p* = 0.0004) and also decreased the decay constants measured from individual slices, especially after 3–5 h of incubation (two-way ANOVA, interaction: *F*_(3,33)_ = 1.4, *p* = 0.26; treatment: *F*_(1,33)_ = 14.8, *p* = 0.0005, Sidak's test, 0.1 vs 3–5 h, *p* = 0.003). ***F***, Significant reduction in the number of colocalized puncta (PI^+^ and GCaMP-filled) across tissue depth with MDL (two-way ANOVA, interaction: *F*_(9,369)_ = 2.17, *p* = 0.023; treatment: *F*_(1,41)_ = 4.19, *p* = 0.047). Inset, significant reduction in total colocalized puncta in individual slices with MDL (Mann–Whitney test, *U* = 86). ***G***, MDL treatment reduced the number of GCaMP-filled neurons across different depths (two-way ANOVA, interaction, *F*_(9,369)_ = 1.46, *p* = 0.038; treatment, *F*_(1,41)_ = 2.8, *p* = 0.01) and total GCaMP-filled neurons per slice (inset, unpaired *t* test). Mean ± 95% CI (median ± IQR for panel ***F*** inset). Using an isoform-specific inhibitor, we found that calpain-2 significantly contributes to the inherent trauma-induced injury in acute brain slices (Extended Data Fig. 6-1). Scale bar, 100 µm.

Our results thus far suggest a functional relationship between trauma and cell death and elevated neuronal Ca^2+^. Persistently high intracellular Ca^2+^ levels can activate calpains, a family of Ca^2+^-dependent proteases mediating various cellular functions ranging from synaptic plasticity to cell death (Baudry and Bi, 2016; Cheng et al., 2018). Previous findings in cultures suggest that Ca^2+^-dependent calpain activity following neuronal hyperexcitability alters nuclear permeability (Bano et al., 2010; Yamashita et al., 2017), which can result in the translocation of cytoplasmatic GCaMPs into the nucleus, rendering neurons “GCaMP-filled” (Suryavanshi et al., 2024). Therefore, we explored whether calpain-mediated mechanisms contribute to trauma-/incubation-induced cell death. After 30 min recovery following slice preparation, we incubated the slices in aCSF containing a nonselective calpain inhibitor (MDL-28710, 30 µM) or vehicle for the duration of the experiment. We found that calpain inhibition significantly reduced the number of PI^+^ cells across different depths (Fig. 6*A*,*B*) and resulted in faster exponential decays of PI^+^ cells across the slice depth, even after prolonged incubation (Fig. 6*C*). There was also a significant decrease in the total number of PI^+^ cells and decay constants with MDL (Fig. 6*D*,*E*). Under conditions of calpain inhibition, we also saw a significant reduction in colocalized puncta (Fig. 6*F*) and the number of GCaMP-filled neurons across slice depth (Fig. 6*G*). These findings suggest that activation of calpain-mediated processes contributes to trauma-/incubation-induced cell death in the neocortex of acute neonatal brain slices.

10.1523/ENEURO.0007-24.2024.f6-1 Fig 6-1 **Calpain-2 isoform significantly contributes to the inherent injury in acute brain slices. A)** Experimental design showing acute slice incubation with nonspecific or a calpain-2 specific inhibitor (MDL and Cal 2-1 respectively) for 2-5 hrs. **B)** *Left:* Normalized PI^+^ cells across multiple depths show faster exponential decays with MDL and Cal 2-1 incubation than aCSF (τ_aCSF_: 48, τ_MDL:_ 22, τ_Cal 2-1:_ 39). *Right:* Both MDL and Cal 2-1 reduced the number of PI^+^ cells after 2-5 hr incubation (One way ANOVA on log-transformed data, F(2, 30) = 4.65, p = 0.017. Tukey’s test for multiple comparisons indicated in figure). **C)** *Left:* Similarly, normalized GCaMP-filled neurons across multiple depths show faster exponential decays with MDL and Cal 2-1 incubation (τ_aCSFP_: 53, τ_MDL:_ 35, τ_Cal 2-1:_ 27). *Right:* Cal 2-1 treatment did not change total GCaMP-filled neurons cells after 2-5 hr incubation (One way ANOVA on log-transformed data, F = (2, 31) = 5.79, p = 0.007 with Tukey’s test for multiple comparison indicated in the figure). Data represented as mean ± 95% CI. Download Fig 6-1, TIF file.

**Movie 1.** Evoked transient Ca^2+^ activity in “healthy” and GCaMP-filled neurons. An example of transient Ca^2+^ activity evoked by a single NMDA puff (125 µM, 100 ms, 3 psi). The movie is split into raw signals (left, pseudocolor) and Δ*F* / *F*_0_ projection (right). Arrows indicate GCaMP-filled neurons. Notice that dark areas on the right during NMDA puffs correlate with filled neurons on the left. Data were acquired at 2.67 Hz. A median filter was applied (radius, 2) for clarity. Playback speed: 24×. Scale bar (white), 100 μm. [View online]

**Movie 2.** Seizure-associated Ca^2+^ activity in “healthy” and GCaMP-filled neurons. Example of neuronal Ca^2+^ transients associated with seizure-like activity induced in acute slices by 4-AP application (100 µM, ∼20 min). The movie is split into raw signals (left, in pseudocolor) and Δ*F* / *F*_0_ projection (right). Arrows indicate GCaMP-filled neurons. Data were acquired at 2.67 Hz. A median filter was applied (radius, 2) for clarity. Playback speed: 24×. Scale bar (white), 100 μm. [View online]

**Movie 3.** Synchronous Ca^2+^ activity associated with experimentally induced seizures in superficial and deeper acute slice tissue. Example of neuronal Ca^2+^ transients associated with seizure-like activity induced in acute slices by 4-AP application (100 µM, 30 min). Ca^2+^ transients in the superficial tissue of the acute slice are displayed on the left, while Ca^2+^ transients in deeper tissue are displayed on the right. Data were acquired at 2.67 Hz. A median filter was applied (radius, 2) for clarity. Playback speed: 24×. Scale bar (white), 100 μm. [View online]

Two well-studied calpain isoforms are expressed in the brain (calpain-1 and -2; Y. Wang et al., 2013; Singh et al., 2014). While synaptic NMDAR-coupled calpain-1 activation promotes long-term hippocampal potentiation, extrasynaptic NMDAR-coupled calpain-2 has been linked to neurodegeneration (Y. Wang et al., 2013; Baudry and Bi, 2016). However, some reports also implicate calpain-1 in ischemic neuronal injury (Cao et al., 2007). To determine the contribution of the calpain-2 isoform, we preincubated PI-loaded Thy1-GCaMP6s slices with a calpain-2-specific inhibitor (Calpain-1 EC_50_: 1,130 nM, Calpain-2 EC_50_: 44 nM; Y. Wang et al., 2014; Baudry et al., 2016, 2024) or with a nonspecific calpain inhibitor (0.5 µM Cal 2-1 or 30 µM MDL-28170; Extended Data Fig. 6-1*A*). Compared with control aCSF, calpain-2 inhibition significantly reduced the total number of PI^+^ cells and the fraction of PI^+^ cells in deeper tissue yet was not different than when using the broad blocker (Extended Data Fig. 4-1*B*). Cal 2-1 preincubation also decreased the GCaMP-filled neurons’ exponential decay but did not change the total number of GCaMP-filled neurons (Extended Data Fig. 4-1*C*). Although the contribution of other isoforms cannot be completely ruled out, our findings suggest that the calpain-2 isoform significantly contributes to trauma-/incubation-induced cell death in acute slices downstream of persistently high [Ca^2+^]_i_.

## Discussion

We explored the spatiotemporal extent of trauma injury in acute neonatal brain slices using PI and Ca^2+^ imaging as markers for cytotoxicity. We observed the following: first, trauma-induced damage was more prominent on the surface of the brain slices and exhibited a depth-dependent decrease (Fig. 1). Second, a spatial correlation exists between cell death and GCaMP-filled neurons across different depths (Fig. 2). Third, prolonged incubations exacerbated the injury in deeper tissue (Fig. 3). Fourth, GCaMP-filled neurons do not participate in network activity (Fig. 4), and there are depth-dependent alterations in seizure-induced neuronal Ca^2+^ responses (Fig. 5), showing that trauma-induced injury alters neuronal responses. Finally, pharmacological inhibition of calpain during incubation mitigated trauma-induced injury and cell death, even during prolonged incubation (Fig. 6). Our results point to a calpain-mediated process downstream of Ca^2+^ entry as a contributing factor for trauma-induced cell death and injury, rendering some neurons nonparticipants in network activity.

This study has certain limitations. First, our scope was limited to neonatal brain slices (P8–12). We did not expand our research to adult slices to determine if cell injury has a similar spatiotemporal evolution as neonatal ones. Second, while recent studies suggest that the viability of acute brain slices can be improved with modified cutting and incubation buffers (Kirov et al., 2004; Hájos and Mody, 2009; Ting et al., 2018; Djurisic, 2020; Perumal and Sah, 2022) or by constant aCSF recirculation through a UV filtration system (Buskila et al., 2014), we did not systematically evaluate this, as this was beyond the scope of this study. Studying every variable is onerous in time and resources. Instead, researchers may want to determine the extent of trauma-induced injury and viability of slices prepared using their protocols, at least with straightforward PI imaging, or take notice of the depth in a slice where the analysis is being conducted to increase rigor and reproducibility. Third, we did not evaluate the molecular signature of the PI^+^ cells without GCaMP6s expression to determine their lineage. This identification was outside our goal, and future studies exploring molecular signatures of these PI^+^ cells, either in fixed tissue or live brain slices, may address their identity. Finally, the imaging resolution was limited for detecting fluorescent signals within the somatic compartments. It was not intended to resolve physiological or morphological features of neurites, as the aim of our study was not to evaluate dendritic compartments. Future studies will address the spatiotemporal extent of neurite injury in acute slices.

Since their inception in the 1960s, acute brain slice preparations remain a critical and popular model in neuroscience research (Yamamoto and McIlwain, 1966; Dingledine et al., 1980; Collingridge, 1995). Acute brain slices are also used to model neurological disorders like epilepsy, stroke, and ischemic and traumatic brain injury (Jarvis et al., 2001; Aiba and Shuttleworth, 2012; Dzhala et al., 2012; Juzekaeva et al., 2020; Suryavanshi et al., 2022; Takezawa et al., 2023). Significantly, traumatic injury during slice preparation can induce long-lasting alterations in neuronal morphology, synaptic activity, and chloride concentrations (Reid et al., 1988; Kirov et al., 1999; Davies et al., 2007; Dzhala et al., 2012; Glykys and Staley, 2016; Glykys et al., 2017). Although most cytotoxic injury is localized to the surface of acute brain slices, recording depths are not commonly described when slices are used for physiological recordings. Also, inherent trauma-induced injury in acute brain slices can significantly confound measurements of neuronal activity caused by perturbations that model neurological disorders. Thus, experiments exploring morphological anomalies or synaptic physiology in acute brain slices are especially susceptible to inconsistent or inaccurate results if the inherent traumatic injury during the preparation of brain slices is not accounted for. Therefore, knowing the spatiotemporal extent of traumatic injury is critical when using acute brain slices.

Estimating trauma injury in slices is challenging because of procedural inconsistencies between preparation paradigms, which vary according to the specific experimental requirements or laboratory protocols. Acute brain slices of various thicknesses (ranging from 200 to 450 µm) are cut in different planes (coronal, horizontal, thalamocortical, etc.) using vibrating blade microtomy or fixed blade tissue slicers, which can cause various degrees of shear stress (Schurr et al., 1985; Reid et al., 1988; Agmon and Connors, 1992; Lein et al., 2011). Brain slice thickness can alter the efficacy of oxygen diffusion, affecting oxidative metabolic burden, which may dictate susceptibility to hyperexcitability or trauma-induced injury (Lindquist and Shuttleworth, 2014). Moreover, extrinsic conditions, like the composition of the aCSF solution and bath temperature during the preparation and incubation (or recovery), may vary between paradigms (Kirov et al., 2004; Ting et al., 2018), and bacterial growth can cause an increase in endotoxins (Terenzi et al., 1995; Buskila et al., 2014; P. Wang et al., 2019). Developmental age also plays a critical role, as acute brain slices prepared from younger animals are more resilient to injury (Ballanyi, 2012; Ting et al., 2014). In our hands, using neonatal acute brain slices, we found that 75% of injured cells were localized to the zone extending from the exposed surface to a depth of ∼90 µm, increasing to a depth of ∼125 µm after 3–5 h of incubation.

Massive tissue trauma during acute slice preparation mimics severe traumatic brain injury (penetrating injury), resulting in cell death. Trauma in acute brain slices, besides causing physical injury, can also result in neuronal hyperexcitability and elevation in intracellular Ca^2+^ (Sun et al., 2008; Szydlowska and Tymianski, 2010). Persistently elevated neuronal Ca^2+^ leads to calpain activation and a cascade of downstream processes, including degradation of nuclear pore complexes and membranes during cell death (Sugiyama et al., 2017; Yamashita et al., 2017) and the enlargement of the nuclear pore complex, leading to the translocation of cytosolic GCaMP into the nucleus (Suryavanshi et al., 2024). We showed that trauma to neonatal brain slices increased the number of PI^+^ cells and GCaMP-filled neurons, caused in part by a calpain-mediated process, as we observed that inhibiting calpains during incubation mitigated cell death and neuronal Ca^2+^ overload in acute brain slices, even after prolonged incubations. Similar findings were observed when using a specific calpain-2 pharmacological inhibitor, suggesting that calpain-2 mediates most trauma-/incubation-induced cell death in acute brain slices. However, as calpain inhibition only partially prevented cell death during slice incubation, our results suggest that other calpain-independent mechanism also mediates cell death during slice trauma. These other mechanisms may include apoptosis, necrosis, necroptosis, and/or ferroptosis, as these have also been implicated in traumatic brain injury (Engel et al., 2011; Bragin et al., 2012; Piao et al., 2012; Vanden Berghe et al., 2014; Liu et al., 2016; Fricker et al., 2018). Hence, based on our results, inhibiting calpain activity may be a potential strategy to improve cellular viability after acute brain trauma, especially during the initial acute clinical presentation (Brorson et al., 1995; Rami et al., 2000; Bevers et al., 2009; Y. Wang et al., 2018).

Finally, we demonstrate that GCaMP6-filled neurons do not participate in network activity and that seizure-related Ca^2+^ dynamics are altered within neocortical circuits in regions with many filled neurons. “Injured” superficial tissue exhibited less frequent and less prominent seizure-related Ca^2+^ spikes than “healthier” deeper tissue in the same slice. These findings indicate that a high number of injured neurons can alter network activity and disease-relevant physiological measurements.

In conclusion, we demonstrate spatiotemporal dynamics of cell injury in acute neonatal brain slices using PI labeling and neuronal Ca^2+^ load as markers for cytotoxicity. We show that trauma leads to cell death (PI^+^), increased GCaMP-filled neurons, and elevated neuropil Ca^2+^, which predominate in the superficial areas of the brain slices. These GCaMP-filled neurons do not participate in network activity and alter seizure dynamics. Blocking calpains decreased cellular death and the number of GCaMP-filled neurons, providing mechanistic insight and a potential strategy to prevent cell death after penetrating brain trauma.

## Data Availability

This study neither used nor generated any new, unique reagents. The datasets and code used for image processing and data analysis in the current study have not been deposited in a public repository but are available on request.

## Synthesis

Reviewing Editor: Jennifer Dulin, Texas A&M University Decisions are customarily a result of the Reviewing Editor and the peer reviewers coming together and discussing their recommendations until a consensus is reached. When revisions are invited, a fact-based synthesis statement explaining their decision and outlining what is needed to prepare a revision will be listed below. The following reviewer(s) agreed to reveal their identity: Michel Baudry. Note: If this manuscript was transferred from JNeurosci and a decision was made to accept the manuscript without peer review, a brief statement to this effect will instead be what is listed below.

This manuscript reports a spatiotemporal characterization of calpain-mediated cell death in acute brain slices from postnatal mouse somatosensory cortex. The authors utilized calcium imaging in parallel with propidium iodide as a measure of cell death to characterize cytotoxicity over space and time in the acute brain slices. While the reviewers agreed that the data are convincing, a major limitation of the manuscript is that it does not represent a major advance in the field nor does it provide significantly new information. The reviewers believed there may be missed opportunities to provide new information, for example about the level of detail regarding mechanism of the calpain-associated cell death. For these reasons, the experiments and subsequent extra analysis needed to revise this paper are substantial and preclude publication in eNeuro.

Reviewer comments:

Reviewer #1:

This technical manuscript details experiments on calcium and cell death imaging of neonatal mouse brain acute slices under endogenous baseline conditions. They image cell death by PI and intracellular calcium with transgenic expression of GCaMP6s. A calpain inhibitor was used to block the baseline pathology.

This paper has key strengths. Admitting and openly addressing the limitations and potential artifact of an experimental system is rare. The authors do this in this work, and they try to resolve it. The study entailed a lot of work and analysis. The statistical table is good.

The manuscript has some weaknesses that could be considered.

1. The age range of the neonatal mice used seems to be large (P8-P12). It should be clearly stated if there were any age effects or if the brain slices from the different ages of mouse had uniform responses. Were the P8 brain slice responses similar to the P12 brain slice responses?

2. There seems to be a lot of temperature variation in the method. The slices were cut in ice-cold aCSF. Then holding at room temp. Then increased to 30C. How did these temperature manipulations affect the results? Were heat shock responses induced?

3. How often was the vibratome blade changed?

4. The authors should rationalize cutting the slices at 450 µm. This thickness is at the limit of effective diffusion of media/solutions. Thicknesses of 200-300 µm might be better.

5. Did slice thickness affects the results?

6. Deeper than 100 µm does no give much of a result. Is that a true negative or a false negative?

7. The authors should describe the kinetics of the GCaMP6s signal. What is the reversibility of the signal? If the signal is not reversible, how is it known whether intracellular calcium is being measured in real time? The authors need to do positive control experiments with calcium ionophores and imaging in real time to what signal rise and signal dissipation.

8. While there is some proximal dendrite labeling for calcium, in general the neuritic labeling is not great. This is a limitation. Why aren't more dendrites labeled. Please explain.

9. The analysis is incremental at different thickness depths. Why are the line graphs continuous?

10. There seems to be layer vulnerability in some of the figures. Is this the case?

Reviewer #2:

This manuscript reports some new data regarding the role of calpain activation in cell death in acute hippocampal neonatal slices. The authors found a depth- and time-dependent cell death, which correlated with increased calcium levels. They also show that treatment with a non-selective calpain inhibitor provided some protection against cell death. They conclude that calpain activation is involved in cell death in acute hippocampal slices.

While the data are quite convincing, the manuscript does not really provide any significantly new information. As the authors indicate, it is well known that acute hippocampal slices exhibit a significant degree of cell death in the outside layers of the slices, and that calcium increase in these cells is critical for cell death. Likewise, the role of calpain activation in cell death is well documented. The authors missed the opportunity to provide really new information regarding several unanswered questions.

1) Which calpain isoform is involved in cell death? We now know that calpain-1 and calpain-2 perform opposite functions in cell death.

2) Which types of cells is degenerating? Are astrocytes, excitatory neurons or inhibitory neurons equally degenerating?

3) What other mechanisms could participate in cell death, since calpain inhibition produces only a partial decrease in cell death?

## Author Response

Dear reviewers, We thank the reviewers for their interest, insightful comments, and suggestions. All the reviewers' comments and concerns (italicized and underlined) are addressed below. We have revised the manuscript based on the reviewers' recommendations.

Based on the reviewer's comments, we included data from new experiments and new analyses of the existing data into 5 new figures (new figures 4 and 5, Extended Data 1-3, 1-4, 1-5, 6-1) and 3 multimedia files (Movie 1-3). The revisions in the main manuscript and statistical table updates are highlighted. However, the small changes in grammar and context to integrate the new findings are not marked.

With your suggestions, our manuscript is much stronger now. We hope that the revised manuscript addresses all your concerns and that you find it favorable.

Respectfully, The authors. ___________ Responses to reviewers' comments:

Reviewer 1 1. The age range of the neonatal mice used seems to be large (P8-P12). It should be clearly stated if there were any age effects or if the brain slices from the different ages of mouse had uniform responses. Were the P8 brain slice responses similar to the P12 brain slice responses? In the updated manuscript, we compared the distribution of PI+ cells and GCaMP-filled neurons across depth between slices prepared from P8-10 and P11-12 mice. We found no statistical difference between them, suggesting no age-related difference in the trauma-induced injury in acute slices (Extended Data 1-4C-D). Manuscript lines (result): 259-265.

2. There seems to be a lot of temperature variation in the method. The slices were cut in ice-cold aCSF. Then holding at room temp. Then increased to 30C. How did these temperature manipulations affect the results? Were heat shock responses induced? The temperatures used for slice preparation and incubation are very well accepted and common for acute brain slice preparations. Cold temperatures during cutting slow down physiological and metabolic processes that contribute to hyperexcitability following trauma. Holding slices at room temperature before returning slices to near-physiological temperatures for recovery mitigates the effects of heat shock that might occur due to rapid temperature rise (Collingridge, 1995; Eguchi et al., 2020; Kirov et al., 2004; Makinson et al., 2017; Thompson et al., 1985; Weilinger et al., 2020). This approach has been common in our laboratory for many years (Three references from our lab 2007, 2009, 2023). Our citations are included in the methods section.

3. How often was the vibratome blade changed? The vibratome blade was changed after every single use. Included in the methods section.

4. The authors should rationalize cutting the slices at 450 µm. This thickness is at the limit of effective diffusion of media/solutions. Thicknesses of 200-300 µm might be better.

We thank the reviewer for bringing up an important issue regarding slice thickness. If there were a diffusion issue for oxygenated aCSF, one would expect more injury in deeper regions, opposite to what we observe. Acute brain slices of 450 µm or thicker are typical for neonatal preparations (Ben-Ari et al., 1989; Dzhala et al., 2012; Glykys et al., 2019; Leinekugel et al., 1997; Takezawa et al., 2023). In the methods section, we now cite previous reports using 450 µm or thicker slices for neonatal preparations. Revised manuscript lines (methods): 105-107.

As suggested, we did new experiments with thinner slices. 350 µm thickness is a typical thickness used for adult brain slice preparations. We now performed additional experiments by preparing 350 µm neonatal slices. We compared the distribution of PI+ cells and GCaMP-filled neurons across depths between 350 and 450 µm thick slices. In 350 µm thick slices, we observed an increase in both PI+ cells and GCaMP-filled neurons in deeper tissue (~300 µm deep, Extended Data 1-4A-B), possibly due to its proximity to the opposite surface of the slice, which received comparable trauma-induced injury as the top surface. Revised manuscript lines (Results): 259-265.

5. Did slice thickness affect the results? Please see the response above.

6. Deeper than 100 µm does no give much of a result. Is that a true negative or a false negative? Significant reduction in PI+ cells and GCaMP-filled neurons beyond 100 µm is a true negative result. The laser power was linearly increased throughout the Z-stack acquisition (16 to 109 mW) to offset the scattering or absorption of photons in deeper tissue. We successfully measured transient neuronal Ca2+ activity at depths of 100 µm in neonatal Thy1-GCaMP6s slices (Figure 5). In our new Extended Figure 1-4, one can see an increase in PI+-cells and GCaMP-filled neurons at 300 microns in the 350 µm thick slices. Also, see Fig. 3B showing an increase in the number of PI+-cells after 3-5 hours in tissue deeper than 200 µm. Thus, the absence of PI+ cells / GCaMP-filled neurons beyond 100 µm tissue can be attributed to the lack of trauma-induced injury, not due to the insufficient fluorophore excitation in deeper tissue (false negative).

7. The authors should describe the kinetics of the GCaMP6s signal. What is the reversibility of the signal? If the signal is not reversible, how is it known whether intracellular calcium is being measured in real time? The authors need to do positive control experiments with calcium ionophores and imaging in real time to what signal rise and signal dissipation.

We have performed additional experiments to describe the difference in the kinetics of GCaMP6s signals between "healthy" neurons and GCaMP-filled neurons. GCaMP-filled neurons show a tonically high fluorescence due to higher baseline Ca2+ loads. Unlike "healthy" neurons, they also lack transient Ca2+ activity following external stimuli (125 µM NMDA puff, 100 ms) or disease-relevant perturbations (seizure-like activity with 100 µM 4-AP, Fig. 4). Of note, GCaMP6s has been widely used to study neuronal activity in vivo and in vitro, and its kinetics and of other GCaMPs have been widely characterized in the past (Chen et al., 2013, PMID: 23868258). Revised manuscript lines (Results): 298-307.

8. While there is some proximal dendrite labeling for calcium, in general the neuritic labeling is not great. This is a limitation. Why aren't more dendrites labeled. Please explain.

The slices were prepared from transgenic mice that express GCaMP6s in neuronal subpopulations under the Thy1 promoter, where GCaMP6s is expressed in somatic and dendritic domains alike, sparsely (Chen et al., 2012; Dana et al., 2014). GCaMP6s was not developed as a morphological marker for dendrites, yet neurites that occupy the neuropil can exhibit strong signals with sufficient elevation in [Ca2+]i. This is reflected in elevated neuropil Ca2+ signal localized to regions with higher neuronal Ca2+ load and cell death (Figure 2). However, accurate morphological and physiological representation of dendrites can be challenging due to insufficient spatial and temporal resolution. We acquired our Z-stacks only at 1X magnification (512x512 pixels) at 0.8 Hz, which is not ideal for imaging dendritic Ca2+ transients, as the aim of our study was not to evaluate dendritic Ca2+ dynamics. This has been clarified in the limitation section (Discussion: 383-387) 9. The analysis is incremental at different thickness depths. Why are the line graphs continuous? The plots showing the normalized or total number of PI+ cells and GCaMP-filled neurons across depth are not cumulative. Rather, each tick on the x-axis represents a 30 μm wide bin. The Z stacks were transformed into maximum intensity projections (MIPs). Each data point represents the number or fraction of PI+ cells or GCaMP-filled neurons in MIPs of 30 µm depth. We apologize for the lack of clarity and have updated the manuscript to provide an appropriate explanation. Revised manuscript lines (methods): 197-199.

10. There seems to be layer vulnerability in some of the figures. Is this the case? To address this, we analyzed the existing data to detect layer-specific localization of both PI+ cells and GCaMP-filled neurons (Extended Data 1-3). We found that the density of PI+ cells and GCaMP-filled neurons was slightly higher in deeper layers. However, we found no statistically significant difference between deeper and superficial layers. Revised manuscript lines (Results): 256-259.

Reviewer #2:

While the data are quite convincing, the manuscript does not really provide any significantly new information. As the authors indicate, it is well known that acute hippocampal slices exhibit a significant degree of cell death in the outside layers of the slices and that calcium increase in these cells is critical for cell death. Likewise, the role of calpain activation in cell death is well documented. The authors missed the opportunity to provide really new information regarding several unanswered questions.

Thank you for your comment as it has allowed us to further clarify our aims and findings in the updated manuscript. We agree that it is well known that the surfaces of acute brain slices are injured following trauma experienced during their preparation. However, the goal of our study is to characterize the spatio-temporal extent of trauma-induced cell death in neonatal acute slices. Second, while calpain activation in cell death is well documented, the majority of prior studies used cultured neurons, which poses a limitation (Bevers et al., 2009; Bevers and Neumar, 2008; Brorson et al., 1995; Cao et al., 2007; Cheng et al., 2018; Sugiyama et al., 2017). Third, other researchers have used different conditions to improve acute brain slices' health, including adding sucrose, low extracellular sodium, etc., and we now show that blocking calpains also improves slices' health, highlighting a more mechanistic reason for neuronal injury after slicing. Fourth, knowing where to conduct physiological recordings (deeper tissue) is especially important since inherent traumatic injury in acute brain slices can significantly confound measurements of neuronal physiology following disease-relevant perturbations. Fifth, we show a strong spatial correlation between cell death and persistent Ca2+ load in neurons and neuropil, and we now show, per reviewers' suggestions, that GCaMP-filled neurons with persistently elevated Ca2+ do not participate in the network activity (Figure 4), even though they are not dead.

In the revised manuscript, we tested if inherent trauma-induced injury altered seizure-related Ca2+ dynamics in neocortical circuits. Seizure-like activity was induced by applying 4-AP (100 µM) in acute slices, and neuronal Ca2+ transients were imaged at "injured" superficial tissue and "healthier" deeper tissue (Figure 5A-B). 4-AP-driven synchronous neuronal Ca2+ transients had significantly lower frequencies, amplitudes, and areas under curve in injured superficial tissue (Figure 5C-F). These results strongly suggest that inherent trauma-induced injury in slices alters disease-relevant neuronal physiological measurements. Revised manuscript lines (Results): 308-317, (Discussion): 363-364, 445-450.

1) Which calpain isoform is involved in cell death? We now know that calpain-1 and calpain-2 perform opposite functions in cell death.

To determine the contribution of specific calpain isoforms, we pharmacologically blocked calpain-2 with a specific inhibitor, Calpain 2-IN-1(0.5 µM). Calpain-2 inhibition significantly reduced total number of PI+ cells compared to aCSF control and reduced the fraction of PI+ cells in deeper tissue but had no effect on total number of GCaMP-filled neurons (Extended Data 6-1). These findings suggest that the calpain-2 isoform is a significant contributor to trauma/incubation-induced cell death in acute slices downstream of persistently high [Ca2+]i. However, the contribution of other isoforms cannot be completely ruled out. Revised manuscript lines (Results): 345-354, (Discussion):433-441.

2) Which types of cells is degenerating? Are astrocytes, excitatory neurons or inhibitory neurons equally degenerating? This is indeed a limitation of this study, and we acknowledge it in the discussion section (Revised manuscript lines 380 - 383). We observed a sparse colocalization between PI+ cells and GCaMP-filled neurons (Figure 1E), suggesting that most dead cells have non-neuronal origins. However, experiments evaluating the molecular lineages of PI+ cells are onerous in time and resources, and they are outside the scope of this manuscript. Future studies will aim to address their molecular identities.

3) What other mechanisms could participate in cell death, since calpain inhibition produces only a partial decrease in cell death? Thank you for this question. The preparation of acute brain slices causes massive trauma to the tissue that mimics severe traumatic brain injury. Multiple cell death cascades are implicated in traumatic brain injury, leading to apoptosis, necrosis, necroptosis, ferroptosis, pyroptosis, etc. (Bragin et al., 2012; Engel et al., 2011; Fricker et al., 2018; Liu et al., 2016; Piao et al., 2012). Therefore, we suspect multiple mechanisms contributing to cell death may be at play in acute brain slices. The partial prevention of cell death by calpain inhibition during slice incubation suggests that other rapid Ca2+/calpain independent mechanisms like unregulated necrosis may play a critical role during slice preparation. We have included a brief discussion of potential alternative mechanisms of cell death. Revised manuscript lines (Discussion): 438-441.

References Ben-Ari Y, Cherubini E, Corradetti R, Gaiarsa JL (1989) Giant synaptic potentials in immature rat CA3 hippocampal neurones. The Journal of Physiology 416:303-325.

Bevers MB, Lawrence E, Maronski M, Starr N, Amesquita M, Neumar RW (2009) Knockdown of m-calpain increases survival of primary hippocampal neurons following NMDA excitotoxicity. J Neurochem 108:1237-1250.

Bevers MB, Neumar RW (2008) Mechanistic Role of Calpains in Postischemic Neurodegeneration. J Cereb Blood Flow Metab 28:655-673.

Bragin A, Benassi SK, Engel J (2012) Patterns of the UP-Down state in normal and epileptic mice. Neuroscience 225:76-87.

Brorson JR, Marcuccilli CJ, Miller RJ (1995) Delayed Antagonism of Calpain Reduces Excitotoxicity in Cultured Neurons. Stroke 26:1259-1267.

Cao G, Xing J, Xiao X, Liou AKF, Gao Y, Yin X-M, Clark RSB, Graham SH, Chen J (2007) Critical Role of Calpain I in Mitochondrial Release of Apoptosis-Inducing Factor in Ischemic Neuronal Injury. J Neurosci 27:9278-9293.

Chen Q, Cichon J, Wang W, Qiu L, Lee S-JR, Campbell NR, DeStefino N, Goard MJ, Fu Z, Yasuda R, Looger LL, Arenkiel BR, Gan W-B, Feng G (2012) Imaging Neural Activity Using Thy1-GCaMP Transgenic mice. Neuron 76:297-308.

Cheng S, Wang S, Lei M, Wang Z, Xiong K (2018) Regulatory role of calpain in neuronal death. Neural Regen Res 13:556.

Collingridge GL (1995) The brain slice preparation: a tribute to the pioneer Henry McIlwain. J Neurosci Methods 59:5-9.

Dana H, Chen T-W, Hu A, Shields BC, Guo C, Looger LL, Kim DS, Svoboda K (2014) Thy1-GCaMP6 Transgenic Mice for Neuronal Population Imaging In Vivo. PLOS ONE 9:e108697.

Dzhala V, Valeeva G, Glykys J, Khazipov R, Staley K (2012) Traumatic Alterations in GABA Signaling Disrupt Hippocampal Network Activity in the Developing Brain. J Neurosci 32:4017-4031.

Eguchi K, Velicky P, Hollergschwandtner E, Itakura M, Fukazawa Y, Danzl JG, Shigemoto R (2020) Advantages of Acute Brain Slices Prepared at Physiological Temperature in the Characterization of Synaptic Functions. Frontiers in Cellular Neuroscience 14.

Engel T, Plesnila N, Prehn JHM, Henshall DC (2011) In vivo contributions of BH3-only proteins to neuronal death following seizures, ischemia, and traumatic brain injury. J Cereb Blood Flow Metab 31:1196-1210.

Fricker M, Tolkovsky AM, Borutaite V, Coleman M, Brown GC (2018) Neuronal Cell Death. Physiol Rev 98:813-880.

Glykys J, Duquette E, Rahmati N, Duquette K, Staley KJ (2019) Mannitol decreases neocortical epileptiform activity during early brain development via cotransport of chloride and water. Neurobiology of Disease 125:163-175.

Kirov SA, Petrak LJ, Fiala JC, Harris KM (2004) Dendritic spines disappear with chilling but proliferate excessively upon rewarming of mature hippocampus. Neuroscience 127:69-80.

Leinekugel X, Medina I, Khalilov I, Ben-Ari Y, Khazipov R (1997) Ca2+ Oscillations Mediated by the Synergistic Excitatory Actions of GABAA and NMDA Receptors in the Neonatal Hippocampus. Neuron 18:243-255.

Liu T, Zhao D, Cui H, Chen L, Bao Y, Wang Y, Jiang J (2016) Therapeutic hypothermia attenuates tissue damage and cytokine expression after traumatic brain injury by inhibiting necroptosis in the rat. Sci Rep 6:24547.

Makinson CD, Tanaka BS, Sorokin JM, Wong JC, Christian CA, Goldin AL, Escayg A, Huguenard JR (2017) Regulation of Thalamic and Cortical Network Synchrony by Scn8a. Neuron 93:1165-1179.e6.

Piao C-S, Loane DJ, Stoica BA, Li S, Hanscom M, Cabatbat R, Blomgren K, Faden AI (2012) Combined inhibition of cell death induced by apoptosis inducing factor and caspases provides additive neuroprotection in experimental traumatic brain injury. Neurobiol Dis 46:745-758.

Sugiyama K, Aida T, Nomura M, Takayanagi R, Zeilhofer HU, Tanaka K (2017) Calpain-Dependent Degradation of Nucleoporins Contributes to Motor Neuron Death in a Mouse Model of Chronic Excitotoxicity. J Neurosci 37:8830-8844.

Takezawa Y, Langton R, Baule SM, Zimmerman MB, Baek S, Glykys J (2023) Role of NKCC1 and KCC2 during hypoxia-induced neuronal swelling in the neonatal neocortex. Neurobiology of Disease 178:106013.

Thompson SM, Masukawa LM, Prince DA (1985) Temperature dependence of intrinsic membrane properties and synaptic potentials in hippocampal CA1 neurons in vitro. J Neurosci 5:817-824.

Weilinger NL, LeDue JM, Kahle KT, MacVicar BA (2020) Quantitative fluorescence lifetime imaging uncovers a novel role for KCC2 chloride transport in dendritic microdomains. bioRxiv 2020.06.08.139592.
