# Supplementary figures and images for: Trauma in Neonatal Acute Brain Slices Alters Calcium and Network Dynamics and Causes Calpain-Mediated Cell Death
Source: eNeuro. 2024 Jul 4;11(7):ENEURO.0007-24.2024. doi: 10.1523/ENEURO.0007-24.2024 (PMC11232372; doi:10.1523/ENEURO.0007-24.2024)

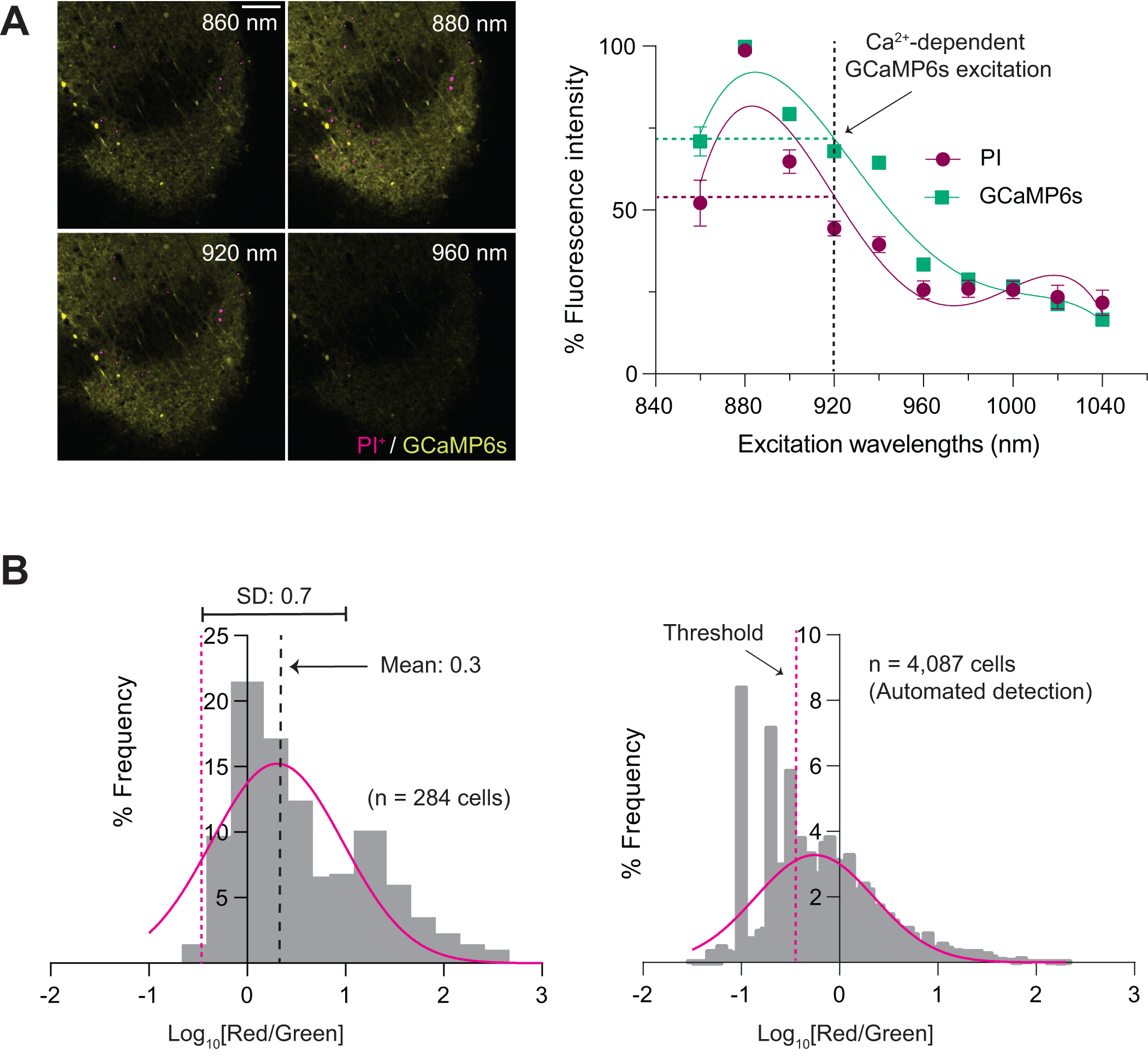

Supplement: Fig 1-1 — Eliminating false PI+ cell detections by emission ratio analysis. A) Left: MIPs showing excitation of PI and GCaMP6 s using multiple 2P wavelengths. Right: Excitation wavelength vs. % emission intensity for PI and GCaMP6 s. There was ∼54% PI emission intensity at 920 nm, a wavelength widely used for Ca2+-dependent GCaMP6 s excitation (n = 8 slices). B) Left: To eliminate false PI + -cell detections due to a green bleed-through signal, a threshold was identified (-0.4, magenta dashed line) from plotting the distribution of Red/Green ratios from a dataset of manually selected “True-positive PI+ cells.” Right: Lognormal distribution of Red/Green ratios of automated PI+ detections across all slices. Detections below the threshold (dashed line) were eliminated as false positives (1,543 cells eliminated). Data represented as mean ± 95% CI. Scale bar = 100 µm. Download Fig 1-1, TIF file. [file eneuro-11-ENEURO.0007-24.2024-s002.tif]

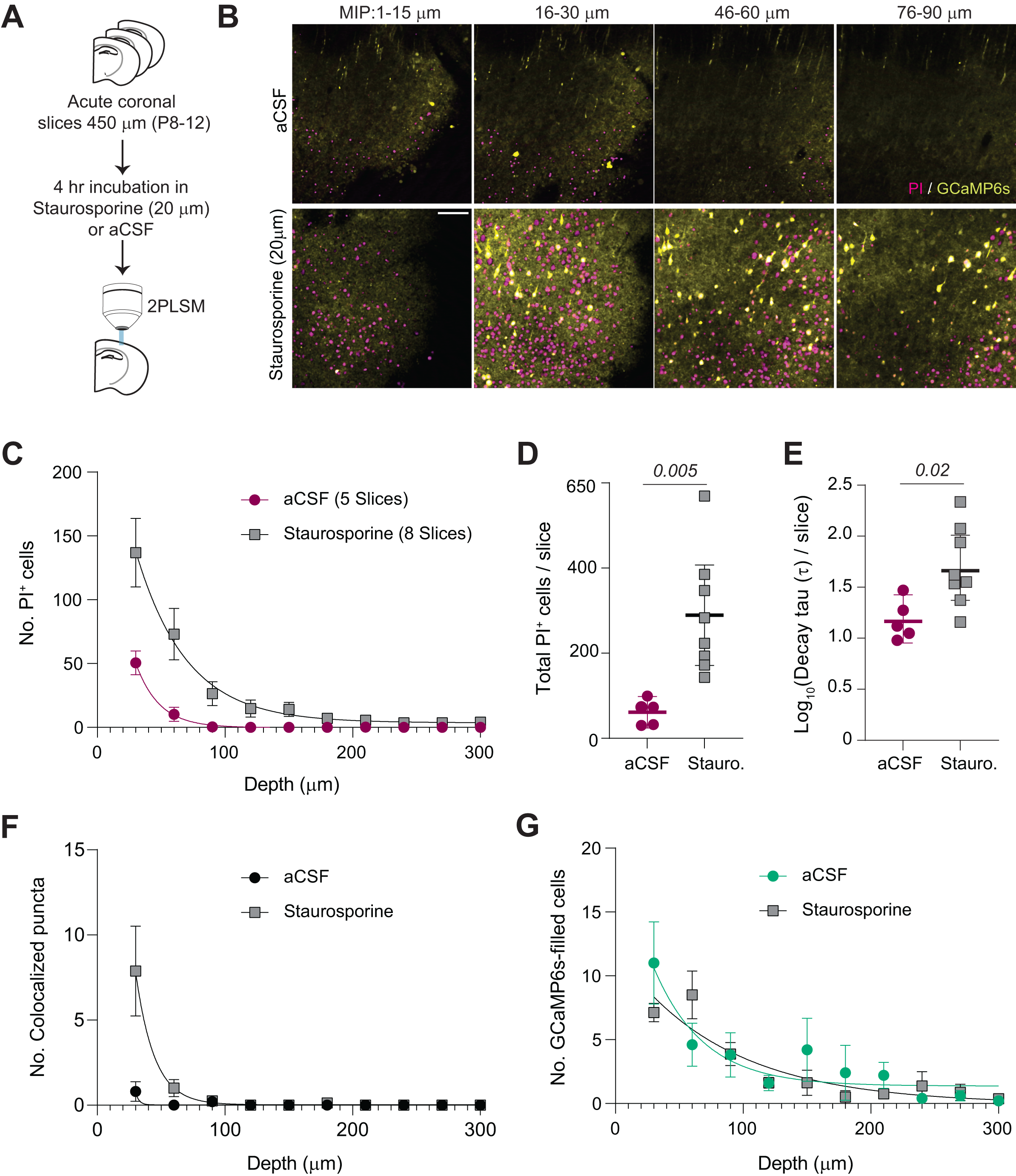

Supplement: Fig 1-2 — Validation of PI + -cell detection across various depths in acute neonatal brain slices. A) Experimental design. B) MIPs representing different depths, acquired from brain slices (post-natal days 8-12), incubated for 4 hours in aCSF alone (top) and staurosporine (20 µM, bottom). C) Staurosporine significantly increased the number of PI + -cells in acute neonatal brain slices across various depths (Two-way ANOVA, interaction: F(9, 99) = 4, p = 0.0002, treatment: F(1, 11) = 12.3, p = 0.0049). D) Increase in the total number of PI + -cells (unpaired t-test, p = 0.005) and (E) in decay constants with staurosporine pre-treatment (unpaired t-test, p = 0.02) calculated from individual slices. F) Increase in colocalized puncta across tissue depth with staurosporine pre-treatment (Two-way ANOVA, interaction: F(9, 99) = 4.15, p < 0.0001, treatment: F(1, 11) = 12.3, p = 0.062). G) Although we found a modestly significant interaction between control and staurosporine-treated slices, post-hoc multiple comparisons confirmed no significant increase in GCaMP6-filled neurons due to staurosporine treatment (Two-way ANOVA, interactions: F(9, 90) = 2.18, p < 0.034, Treatment: F(1, 10) = 0.041, p = 0.93, Sidak’s test for multiple comparisons p > 0.05). aCSF: 5 slices; staurosporine: 8 slices. Mean ± 95% CI. Scale bar = 100 µm. Download Fig 1-2, TIF file. [file eneuro-11-ENEURO.0007-24.2024-s003.tif]

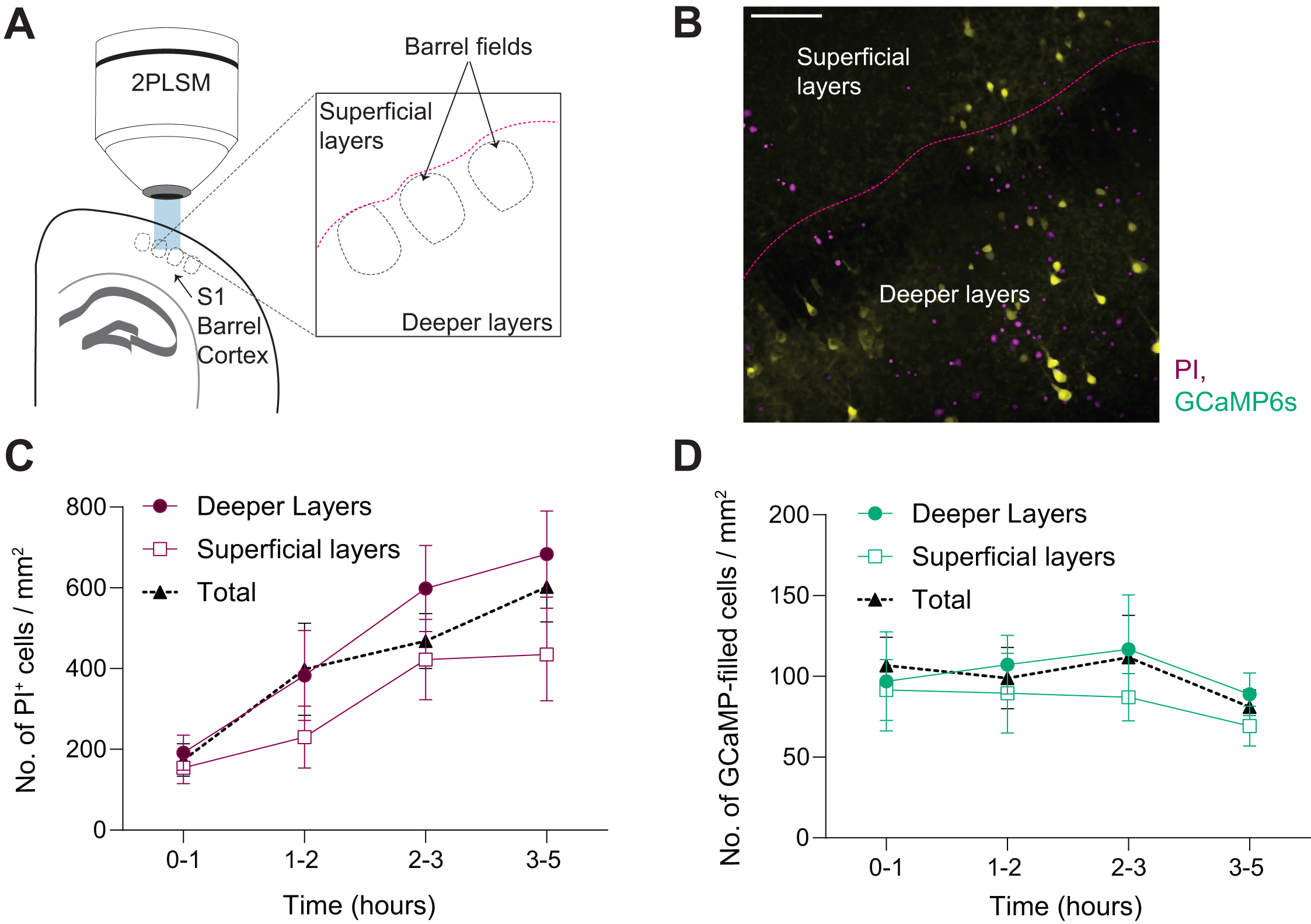

Supplement: Fig 1-3 — Layer-specific distribution of PI+ and GCaMP-filled neurons A) Experimental design describing a strategy for imaging superficial and deeper layers of the primary somatosensory cortex. B) Representative image showing PI+ and GCaMP-filled distribution neurons across superficial and deeper layers. C) Density of PI+ cells and D) GCaMP-filled neurons in superficial and deeper layers, measured from acute brain slices at different durations of incubation, showed no significant difference between layers (PI+: Two-way ANOVA, interaction: F(6, 87) = 0.3, p = 0.936; GCaMP-filled: Two-way ANOVA, interaction: F(6, 82) = 0.06, p = 0.99). Data represented as mean ± 95% CI. Scale bar = 100 µm. Download Fig 1-3, TIF file. [file eneuro-11-ENEURO.0007-24.2024-s004.tif]

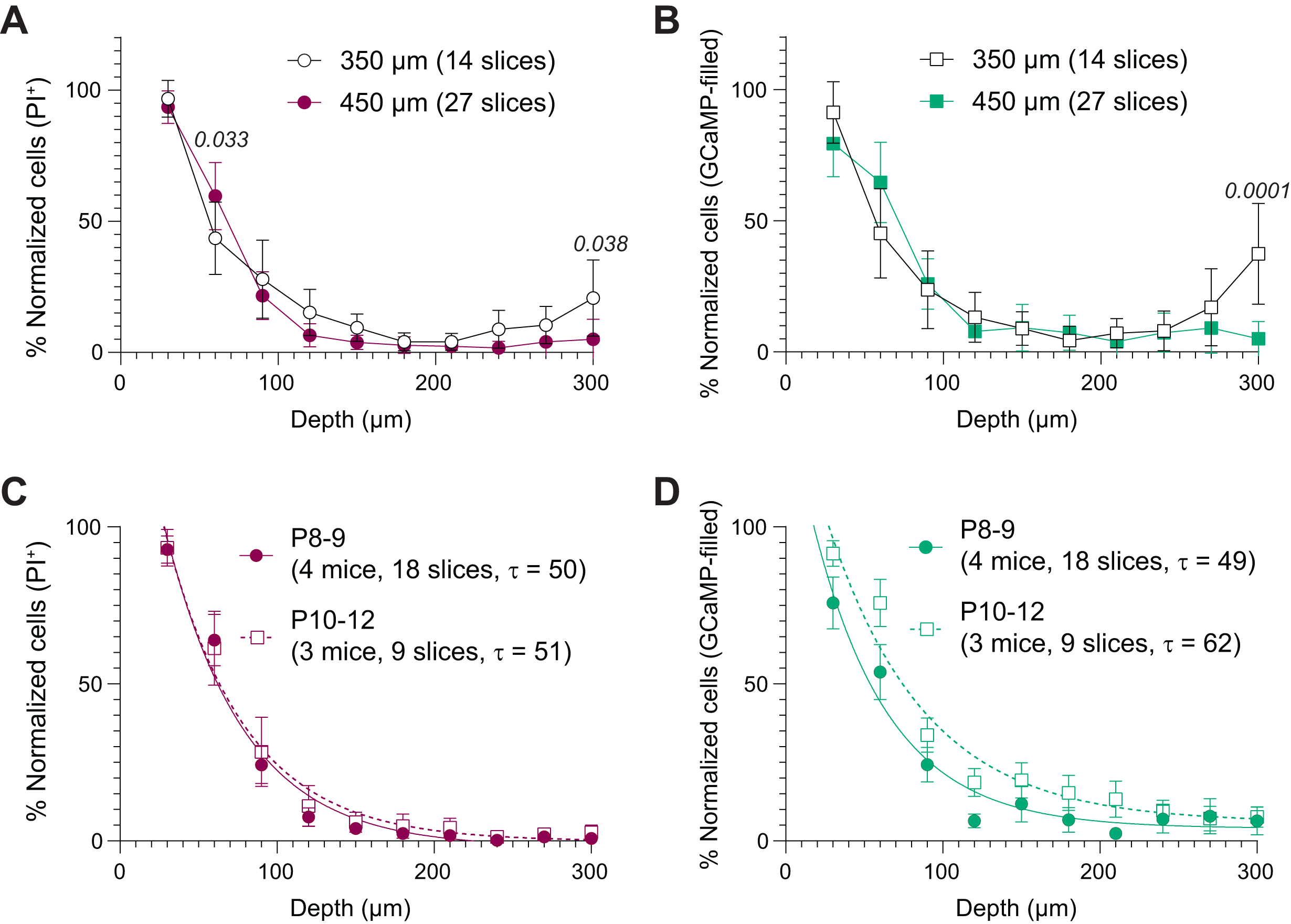

Supplement: Fig 1-4 — Effects of slice thickness and postnatal age on inherent injury in acute brain slices A) Differences in normalized PI+ cells across depths between 350 and 450 µm thick acute brain slices (Two-way ANOVA, Interaction: F(9, 351) = 2.04, p = 0.034), with an increase at 300 µm depth in the 350 µm thick slices (Sidak’s multiple comparisons, p = 0.038). B) Significant difference between the distribution of GCaMP-filled neurons across depths (Two-way ANOVA, Interaction: F(9, 342) = 2.04, p = 0.0007), with an increase at 300 µm in the 350 µm thick slices (Sidak’s multiple comparisons, p = 0.0001). No postnatal age difference in C) PI+ cells (Two-way ANOVA, Interaction: F(9, 216) = 0.08, p = 0.99) or D) GCaMP-filled neurons (Two-way ANOVA, Interaction: F(9, 306) = 0.87, p = 0.56). Data represented as mean ± 95% CI. Download Fig 1-4, TIF file. [file eneuro-11-ENEURO.0007-24.2024-s005.tif]

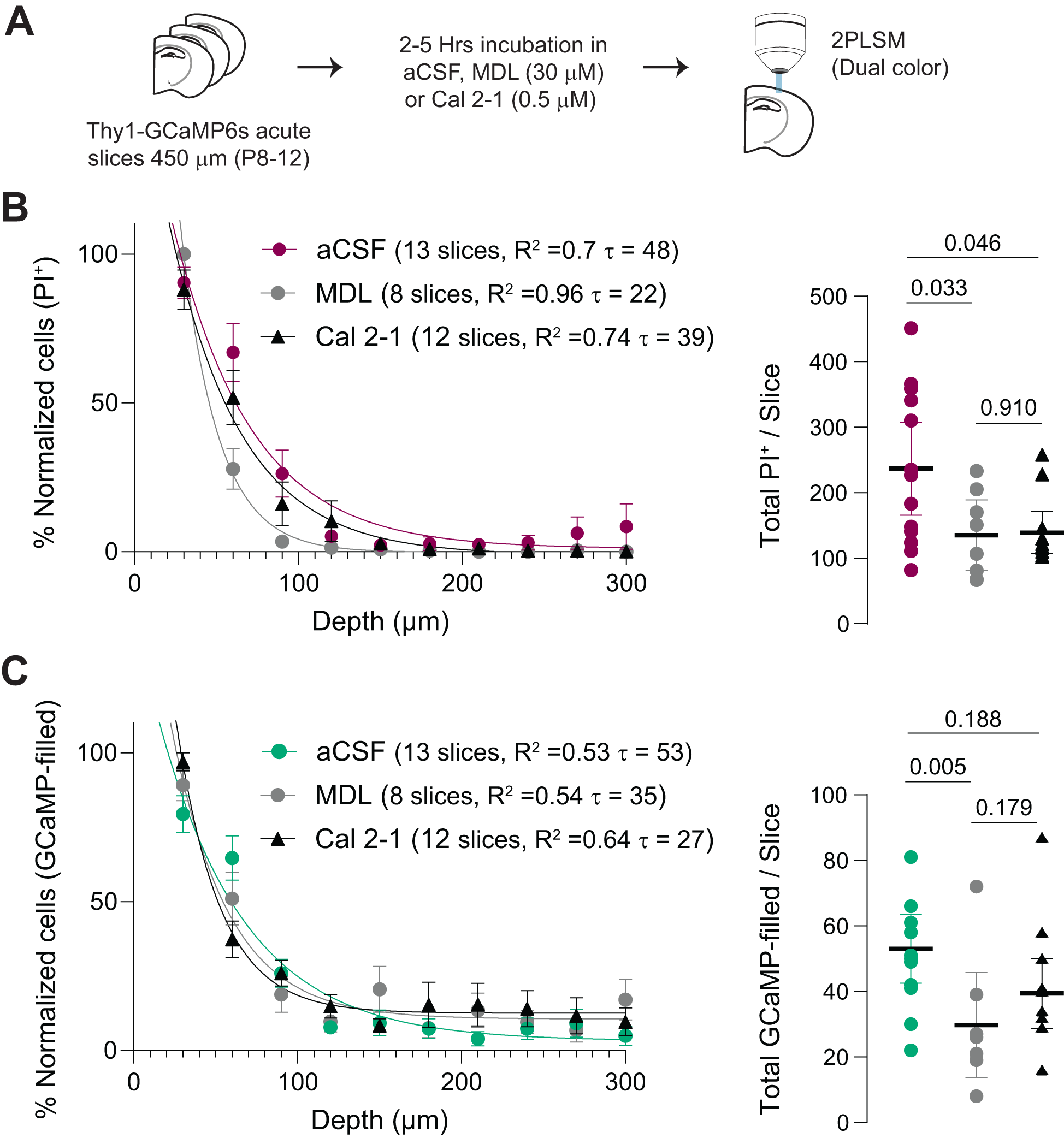

Supplement: Fig 6-1 — Calpain-2 isoform significantly contributes to the inherent injury in acute brain slices. A) Experimental design showing acute slice incubation with nonspecific or a calpain-2 specific inhibitor (MDL and Cal 2-1 respectively) for 2-5 hrs. B) Left: Normalized PI+ cells across multiple depths show faster exponential decays with MDL and Cal 2-1 incubation than aCSF (τaCSF: 48, τMDL: 22, τCal 2-1: 39). Right: Both MDL and Cal 2-1 reduced the number of PI+ cells after 2-5 hr incubation (One way ANOVA on log-transformed data, F(2, 30) = 4.65, p = 0.017. Tukey’s test for multiple comparisons indicated in figure). C) Left: Similarly, normalized GCaMP-filled neurons across multiple depths show faster exponential decays with MDL and Cal 2-1 incubation (τaCSFP: 53, τMDL: 35, τCal 2-1: 27). Right: Cal 2-1 treatment did not change total GCaMP-filled neurons cells after 2-5 hr incubation (One way ANOVA on log-transformed data, F = (2, 31) = 5.79, p = 0.007 with Tukey’s test for multiple comparison indicated in the figure). Data represented as mean ± 95% CI. Download Fig 6-1, TIF file. [file eneuro-11-ENEURO.0007-24.2024-s006.tif]
